# RNA-Seq Data Mining: Downregulation of NeuroD6 Serves as a Possible Biomarker for Alzheimer's Disease Brains

**DOI:** 10.1155/2014/123165

**Published:** 2014-12-08

**Authors:** Jun-ichi Satoh, Yoji Yamamoto, Naohiro Asahina, Shouta Kitano, Yoshihiro Kino

**Affiliations:** Department of Bioinformatics and Molecular Neuropathology, Meiji Pharmaceutical University, 2-522-1 Noshio, Kiyose, Tokyo 204-8588, Japan

## Abstract

Alzheimer's disease (AD) is the most common cause of dementia worldwide with no curative therapies currently available. Previously, global transcriptome analysis of AD brains by microarray failed to identify the set of consistently deregulated genes for biomarker development of AD. Therefore, the molecular pathogenesis of AD remains largely unknown. Whole RNA sequencing (RNA-Seq) is an innovative technology for the comprehensive transcriptome profiling on a genome-wide scale that overcomes several drawbacks of the microarray-based approach. To identify biomarker genes for AD, we analyzed a RNA-Seq dataset composed of the comprehensive transcriptome of autopsized AD brains derived from two independent cohorts. We identified the core set of 522 genes deregulated in AD brains shared between both, compared with normal control subjects. They included downregulation of neuronal differentiation 6 (NeuroD6), a basic helix-loop-helix (bHLH) transcription factor involved in neuronal development, differentiation, and survival in AD brains of both cohorts. We verified the results of RNA-Seq by analyzing three microarray datasets of AD brains different in brain regions, ethnicities, and microarray platforms. Thus, both RNA-Seq and microarray data analysis indicated consistent downregulation of NeuroD6 in AD brains. These results suggested that downregulation of NeuroD6 serves as a possible biomarker for AD brains.

## 1. Introduction

Alzheimer's disease (AD) is the most common cause of dementia worldwide affecting the elderly population, characterized by the hallmark pathology of amyloid-*β* (A*β*) deposition, neurofibrillary tangle (NFT) formation, and extensive neurodegeneration in the brain. The complex interaction between multiple genetic and environmental factors affecting various molecular pathways plays a key role in the pathogenesis of AD [1]. With regard to environmental factors, disturbed homeostasis of dietary metals, such as copper, aluminum, and iron, confers an increased risk of AD [2, 3]. With respect to genetic factors, genome-wide association studies (GWAS), composed of large cohorts of AD and controls, identified numerous common variants but with smaller risks associated with development of late-onset AD [4]. They include complement component receptor 1 (*CR1*), bridging integrator 1 (*BIN1*), clusterin (*CLU*), phosphatidylinositol binding clathrin assembly protein (*PICALM*), membrane-spanning 4-domains, subfamily A, member 4A/membrane-spanning 4-domains, subfamily A, member 6E (*MS4A4/MS4A6E*), CD2-associated protein (*CD2AP*), CD33 molecule (*CD33*), EPH receptor A1 (*EPHA1*), and ATP-binding cassette, subfamily A, member 7 (*ABCA7*) [4]. More recently, whole-exome sequencing (WES) studies discovered rare functional variants located in the genes encoding A*β* precursor protein (*APP*), triggering receptor expressed on myeloid cells 2 (*TREM2*), and phospholipase D3 (*PLD3*), exhibiting a much greater contribution to protection or development of AD [5–7]. However, at present, the central molecular mechanism underlying neurodegeneration in AD remains largely unknown. Therefore, no curative therapies based on the molecular pathogenesis of AD are currently available.

The completion of the Human Genome Project in 2003 allows us to systematically study disease-associated profiles of the whole human genome. Particularly, microarray technologies enable us not only to identify disease-specific molecular signatures and biomarkers for diagnosis and prediction of prognosis but also to characterize druggable targets for effective therapy. Actually, global transcriptome analysis of postmortem AD brains by microarray has identified a battery of genes aberrantly regulated in AD, whose role has not been previously predicted in its pathogenesis [8]. They include reduced expression of kinases/phosphatases, cytoskeletal proteins, synaptic proteins, and neurotransmitter receptors in NFT-bearing CA1 neurons [9], downregulation of neurotrophic factors and upregulation of proapoptotic molecules in the hippocampal CA1 region [10], disturbed sphingolipid metabolism in various brain regions during progression of AD [11], and overexpression of the AMPA receptor GluR2 subunit in synaptosomes of the prefrontal cortex [12]. However, previous studies failed to identify the set of definite biomarker genes, whose expression is consistently deregulated in AD brains across different studies [8]. The failure in reproducibility of the results is attributable to differences in study designs and samples, including the quality of RNA, disease stages, brain regions, cellular diversities, ethnicities, and microarray platforms [13].

Recently, the revolution of the next-generation sequencing (NGS) technology has made a great impact on the field of genome research. Whole RNA sequencing (RNA-Seq) serves as an innovative tool for the comprehensive transcriptome profiling on a genome scale in a high-throughput and quantitative manner [14, 15]. RNA-Seq clarifies the unbiased expression of the complete set of transcripts at a single base resolution, including splice junctions and fusion genes, by providing digital gene expression levels with high reproducibility. RNA-Seq enables us to characterize the complex transcriptome, composed of mRNAs, noncoding RNAs, and small RNAs, theoretically at a single cell level, by aligning sequencing reads on reference genomes or assembling them* de novo* without references. For these reasons, RNA-Seq overcomes several drawbacks intrinsic to the microarray-based approach that is hampered by the difficulty in detection of novel transcripts and splice variants, the poor sensitivity of rare transcripts, and high backgrounds due to cross hybridization.

To identify biomarker genes relevant to the molecular pathogenesis of AD, we analyzed publicly available RNA-Seq datasets, composed of the comprehensive transcriptome of autopsied AD brains derived from two independent cohorts. First, we identified the core set of 522 genes deregulated in AD brains overlapping between both. Then, we verified the results of RNA-Seq by analyzing three independent microarray datasets of AD brains that are different in brain regions, ethnicities, and microarray platforms. Consequently, we found consistent downregulation of neuronal differentiation 6 (NeuroD6), a bHLH transcription factor involved in neuronal development and differentiation, serving as a possible biomarker for AD brains.

## 2. Materials and Methods

### 2.1. RNA-Seq Datasets of AD Brains

To identify a comprehensive set of differentially expressed genes (DEGs) in the brains of AD patients compared with normal control (NC) subjects, we investigated FASTQ-formatted files of RNA-Seq datasets retrieved from the DDBJ Sequence Read Archive (DRA) (https://trace.ddbj.nig.ac.jp/DRASearch) under the accession number of SRA060572. It consisted of 15 separate samples derived from two independent cohorts, studied by the researchers in Emory University, Atlanta, here abbreviated as EMU, and by those in the University of Kentucky, Lexington, abbreviated as UKY [16]. The EMU dataset contains transcriptome of the frontal cortex isolated from three male and two female AD patients with age = 71.0 ± 8.2 years and postmortem interval (PMI) = 13.8 ± 7.2 hours and two male and two female NC subjects with age = 60.8 ± 3.3 years and PMI = 9.0 ± 2.6 hours. The UKY dataset contains transcriptome of the frontal cortex isolated from three female AD patients with age = 81.7 ± 3.2 years and PMI = 2.2 ± 0.4 hours and three female NC subjects with age = 85.0 ± 1.0 years and PMI = 2.8 ± 0.6 hours. The information on the Braak stage of AD pathology [17] is not available for any cases. In these experiments, total RNA was purified by oligo (dT) beads and converted to cDNA for PCR amplification using SMARTer PCR cDNA Synthesis Kit (Clontech). Then, PCR products were fragmented and processed for DNA library preparation via PCR amplification using NEBNext DNA Library Prep Master Mix Set for Illumina (New England BioLabs). The final DNA library products with size ~200 bp were prepared for paired-end sequencing on HiSeq 2000 (Illumina).

After removing poly-A tails and low quality reads from the original data, we mapped short read data on the human genome reference sequence hg19 by using TopHat2.0.9 (http://ccb.jhu.edu/software/tophat/index.shtml). The expression levels were transformed into fragments per kilobase of exon per million mapped fragments (FPKM). We identified DEGs that satisfy the significance expressed as *q*-value representing FDR-adjusted *P* value < 0.05 by using Cufflinks2.1.1 (http://cufflinks.cbcb.umd.edu).

### 2.2. Microarray Datasets of AD Brains

To verify the results of RNA-Seq data analysis, we investigated three distinct microarray datasets of AD brains retrieved from Gene Expression Omnibus (GEO) (http://www.ncbi.nlm.nih.gov/geo/) under accession numbers of GSE1297, GSE5281, and GSE11829. The GSE1297 dataset contains transcriptome of postmortem hippocampal CA1 tissues studied on a Human Genome U133A Array containing 22,215 transcripts (Affymetrix), and the data were normalized by the Microarray Analysis Suite 5.0 (MAS5) algorithm [18]. The samples were collected by the researchers in UKY. They were prepared from 31 age-matched individuals, composed of nine NC subjects (age = 85.3 ± 8.0 years; male = 7, female = 2), seven patients with incipient AD (age = 91.9 ± 6.2 years; male = 2, female = 5), eight with moderate AD (age = 83.4 ± 3.2 years; male = 2, female = 6), and seven with severe AD (age = 84.0 ± 10.6 years; male = 2, female = 5). The clinical stage of AD was defined by the Mini-Mental State Examination (MMSE) score as follows: the control (the score > 25), incipient (20–26), moderate (14–19), and severe (<14) AD. The information on the Braak stage of AD pathology is not available for any cases.

The GSE5281 dataset, alternatively named steph-affy-human 433773, contains transcriptome of laser microdissection (LCM)-captured layer III neurons derived from various brain regions studied on a Human Genome U133 Plus 2.0 Array containing 47,400 transcripts (Affymetrix), and the data were normalized by MAS5 [19]. The samples were collected by the researchers in AD Centers of Arizona, Duke University, and Washington University. We studied the gene expression profile of cortical neurons in the superior frontal gyrus, which were isolated from 11 age-matched NC subjects (age = 79.3 ± 10.2 years; male = 7, female = 4) and 23 AD patients (age = 79.2 ± 7.5 years; male = 13, female = 10). The information on the Braak stage of AD pathology is not available for any cases.

The GSE36980 dataset contains transcriptome of postmortem brain tissues isolated from frontal and temporal cortices and the hippocampus studied on a Human Gene 1.0 ST Array containing 28,869 genes (Affymetrix), and the data were normalized by the robust multiarray average (RMA) algorithm [20]. The samples were collected by the researchers in Kyushu University, Japan, for the Hisayama study. We studied the gene expression profile of the hippocampus, derived from ten non-AD controls (age = 77.0 ± 9.0 years; male = 5, female = 5) and seven AD patients (age = 92.9 ± 6.1 years; male = 3, female = 4). The information on the Braak stage of AD pathology is not available for any cases.

To evaluate the statistically significant difference in gene expression levels between AD and NC or non-AD groups, we performed a two-tailed Welch *t*-test by using TTEST function of Excel. In some experiments, we performed receiver operating characteristic (ROC) analysis by using SPSS version 19 (IBM).

### 2.3. Molecular Network Analysis

We imported Entrez Gene IDs of DEGs into the Functional Annotation tool of Database for Annotation, Visualization and Integrated Discovery (DAVID) v6.7 [21]. DAVID extracts gene ontology (GO) terms enriched in the set of imported genes and identifies relevant pathways constructed by Kyoto Encyclopedia of Genes and Genomes (KEGG). The results are followed by statistical evaluation with the modified Fisher exact test corrected by multiple comparison tests. We considered *P* value < 0.05 after Bonferroni's correction as significant. KEGG is a publicly accessible knowledgebase that contains 337,524 manually curated pathways that cover a wide range of metabolic, genetic, environmental, and cellular processes and human diseases [22].

We also imported Entrez Gene IDs of DEGs into the Core Analysis tool of Ingenuity Pathways Analysis (IPA) (Ingenuity Systems). IPA is a commercial knowledgebase that contains approximately 3,000,000 biological and chemical interactions with definite scientific evidence. By uploading the list of Gene IDs, the network-generation algorithm identifies focused genes integrated in global molecular pathways and networks. IPA calculates the score *P* value that reflects the statistical significance of association between the genes and the pathways and networks by Fisher's exact test. We considered *P* value < 0.05 by Fisher's exact test as significant.

## 3. Results

### 3.1. RNA-Seq Data Analysis of AD Brains

By RNA-Seq data analysis with the combination of TopHat and Cufflinks, we studied transcriptome of the frontal cortex of AD and NC derived from two distinct cohorts named EMU and UKY. We identified 587,301 and 766,998 consensus transcripts in total from datasets of EMU and UKY, respectively. Among them, we identified 1,226 DEGs for EMU and 2,625 DEGs for UKY that satisfy *q*-value (FDR-corrected *P* value) < 0.05 and fold change greater than 2.0 or smaller than 0.5, when compared between AD and NC groups. Then, we extracted the core set of 522 DEGs overlapping between both cohorts, composed of 470 downregulated and 52 upregulated genes in AD (see Supplementary Table  1 of the Supplementary Material available online at http://dx.doi.org/10.1155/2014/123165). Thus, downregulated genes greatly outnumbered upregulated ones in AD brains. Top 20 genes are listed in Table 1. Notably, the expression of neuronal differentiation 6 (NeuroD6), a brain-specific basic helix-loop-helix (bHLH) transcription factor [23], is greatly reduced at fold changes 0.095 for EMU and 0.159 for UKY in AD brains (*q* = 0.0023  for EMU and 0.0006 for UKY) (Table 1, italicized). Furthermore, lipid phosphate phosphatase-related protein type 4 (LPPR4; PRG1), a direct target gene of NeuroD6 [24], was also downregulated in AD brains of both cohorts (Supplementary Table  1). We identified totally 60 differentially spliced genes in the frontal cortex of AD, when the data derived from both cohorts were combined, although none of them were shared between both (Supplementary Table  2).

**Figure 1:**
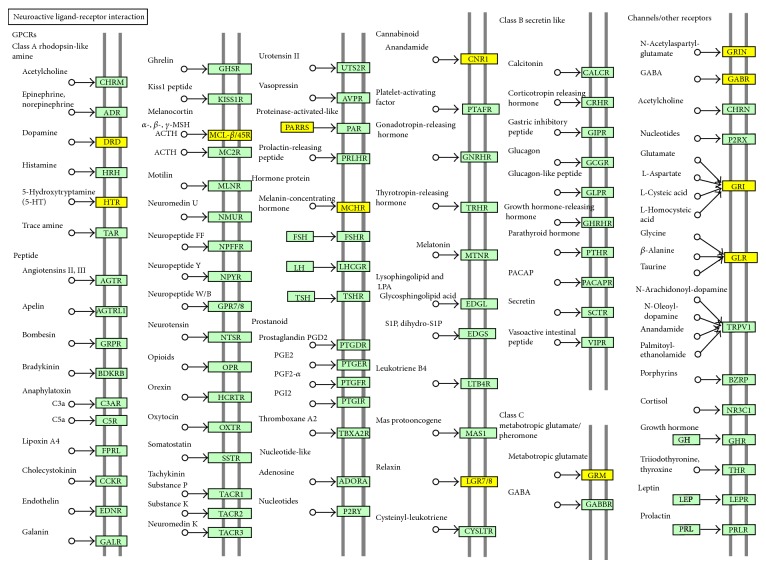
KEGG pathway of 470 DEGs downregulated in the frontal cortex of AD identified by RNA-Seq data analysis. Entrez Gene IDs of 470 DEGs downregulated in the frontal cortex of AD identified by RNA-Seq data analysis of SRA060572 were imported into the Functional Annotation tool of DAVID. It extracted the most significant KEGG pathway termed “neuroactive ligand-receptor interaction” (hsa04080) relevant to the set of imported genes. Downregulated genes are colored yellow.

**Figure 2:**
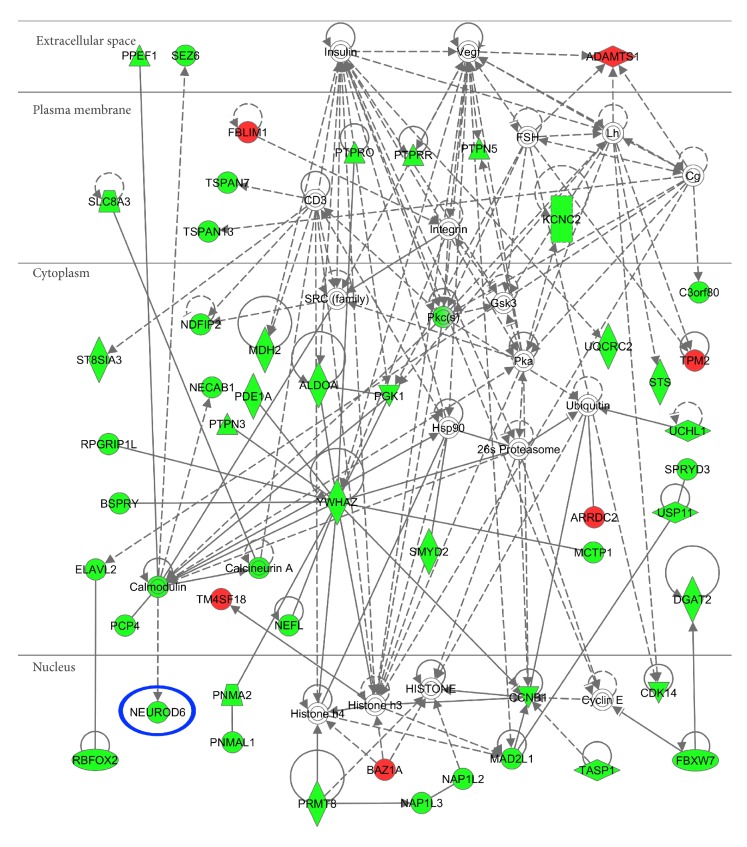
IPA pathways of the core set of 522 DEGs in the frontal cortex of AD identified by RNA-Seq data analysis. Entrez Gene IDs of 522 DEGs in the frontal cortex of AD identified by RNA-Seq data analysis of SRA060572 were imported into the Core Analysis tool of IPA. It extracted the most significant functional network termed “Hereditary Disorder, Neurological Disease, Psychological Disorders” relevant to the set of imported genes. Downregulated DEGs are colored green, while upregulated DEGs are colored red. NeuroD6 is highlighted by a blue circle.

DAVID revealed that the set of 470 DEGs downregulated in the frontal cortex of AD are relevant to GO terms of “synaptic transmission” (GO:0007268; *P* = 2.545*E* − 17 corrected by Bonferroni) and “transmission of nerve impulse” (GO:0019226; *P* = 1.778*E* − 15 corrected by Bonferroni). They are also relevant to the KEGG pathway named “neuroactive ligand-receptor interaction” (hsa04080; *P* = 0.0004 corrected by Bonferroni) (Figure 1). In contrast, the set of 52 genes upregulated in AD were not significantly associated with any GO terms or KEGG pathways. IPA showed that the core set of 522 DEGs have a significant relationship with top two different functional networks defined as “Cell-To-Cell Signaling and Interaction, Nervous System Development and Function, Neurological Disease” (*P* = 1.00*E* − 71) and “Hereditary Disorder, Neurological Disease, Psychological Disorders” (*P* = 1.00*E* − 71) (Figure 2). Taken together, these results suggest that a battery of the genes essential for neuronal interactions is coordinately downregulated in AD brains.

### 3.2. Microarray Data Analysis of AD Brains

To verify the results of RNA-Seq data analysis, we studied three distinct microarray datasets of AD brains numbered GSE1297, GSE5281, and GSE36980. We compared the core set of 522 DEGs of RNA-Seq with DEGs extracted from microarray datasets. First, we studied the GSE5281 dataset composed of transcriptome of LCM-captured cortical neurons in the superior frontal gyrus. We identified the set of 215 DEGs compared between AD and NC groups, including 210 downregulated and 5 upregulated genes in AD (Supplementary Table  3). Because downregulated genes greatly outnumbered upregulated classes, we thereafter focused on the downregulated set. Among them, we found that 15 genes correspond to the core set of 522 DEGs of RNA-Seq (Table 2). Notably, the expression of NeuroD6 was reduced at fold change = 0.238 in purified cortical neurons of the superior frontal gyrus in AD brains (*P* = 0.000066, Table 2, italicized). In contrast, the levels of expression of NeuroD1 were not significantly different between AD and NC brains (*P* = 0.530, not shown). ROC analysis indicated that the area under the ROC curve (AUC) is 0.893 for NeuroD6 and 0.474 for NeuroD1, and the levels of sensitivity and specificity for discrimination between AD and NC are acceptable for NeuroD6 (*P* = 2.494*E* − 04) but unacceptable for NeuroD1 (*P* = 0.811) (Supplementary Figure  1).

Next, we attempted to answer the question whether downregulation of NeuroD6 serves as a possible biomarker for diagnosis of AD by brain transcriptome profiling, regardless of differences in brain regions, microarray platforms, or ethnicities of samples. We analyzed two more datasets of transcriptome of postmortem hippocampal tissues isolated from Caucasian (GSE1297 on Human Genome U133A Array) or Japanese (GSE36980 on Human Gene 1.0 ST Array) AD patients. From the GSE1297 dataset, we identified the set of 131 DEGs downregulated in the hippocampal CA1 region at fold change of severe AD versus NC < 0.6 (Supplementary Table  4). We found that 25 genes of 131 DEGs correspond to the core set of 522 DEGs of RNA-Seq (Table 3). They also included NeuroD6 whose expression levels are reduced in Caucasian AD brains during progression of AD at fold change = 0.569 for the comparison between severe AD and NC (*P* = 0.0072, Table 3, italicized). From the GSE11829 dataset, we identified the set of 31 DEGs downregulated in the region-unrestricted hippocampus of Japanese AD patients, compared with non-AD controls (Supplementary Table  5). We found that 12 genes of 31 DEGs correspond to the core set of 522 DEGs of RNA-Seq (Table 4). Again, they included NeuroD6 whose expression levels are reduced in AD brains at fold change = 0.433 for the comparison between AD and non-AD (*P* = 0.0016, Table 4, italicized). Taken together, these observations suggest that downregulation of NeuroD6 serves as a fairly universal biomarker for diagnosis of AD by brain transcriptome profiling, regardless of differences in brain regions, microarray platforms, or ethnicities of samples.

## 4. Discussion

Previously, a number of microarray-based transcriptome studies of AD brains failed to identify the set of consistently deregulated genes across different studies [8]. RNA-Seq serves as an innovative technology for the comprehensive transcriptome profiling on a genome scale in a high-throughput and quantitative manner [14, 15]. To identify biomarker genes relevant to the molecular pathogenesis of AD, we first studied publicly available RNA-Seq datasets of AD brain transcriptome derived from two independent cohorts named EMU and UKY. We identified the core set of 522 DEGs consistently deregulated in AD brains of both cohorts. They include 470 downregulated and 52 upregulated genes in AD brains, relevant to synaptic transmission, neuroactive ligand-receptor interaction, nervous system development, and pathological processes of neuropsychiatric diseases by GO and pathway analysis. Then, we compared the results of RNA-Seq data analysis with those of three distinct microarray datasets of AD brains, which are different in brain regions, ethnicities, and microarray platforms. As a result, we identified consistent downregulation of NeuroD6 in AD brains throughout the datasets studied.

The NeuroD family of bHLH transcription factors, composed of three major members, such as NeuroD1 (BETA2), NeuroD2 (NDRF), and NeuroD6 (NEX1, MATH2, and ATOH2), acts as a differentiation factor for neural precursor cells in the developing central nervous system (CNS) [23]. Each member exhibits an overlapping but distinct spatiotemporal expression profile with partially redundant function in the formation of subpopulations of neurons. A previous study by* in situ* hybridization showed that NeuroD6 is expressed abundantly in mature adult neurons of the cerebral cortex, the hippocampus, and the cerebellum [25]. Although NeuroD6-deficient mice exhibit no obvious defect in development, NeuroD1/NeuroD6 double knockout mice show arrest of terminal differentiation of granule cells in the hippocampus [26]. NeuroD6-expressing progenitor cells located in the subventricular zone have a capacity to differentiate into pyramidal glutamatergic neurons in upper cortical layers [27]. Both NeuroD2 and NeuroD6 regulate axonal fasciculation and proper formation of callosal fiber tracts [28]. NeuroD6 plays a key role in cell fate decision of subtypes of amacrine cells in the retina [29]. Constitutive expression of NeuroD6 triggers neuronal differentiation of PC12 cells, originated from a pheochromocytoma of the rat adrenal medulla, without requirement of nerve growth factor (NGF) [30]. NeuroD6 plays a decisive role in the switch from proapoptotic to antiapoptotic pathways during neuronal differentiation of PC12 cells [31]. Furthermore, NeuroD6 confers tolerance to oxidative stress by inducing antioxidant responses and by increasing the mitochondrial biomass [32]. Importantly, NeuroD6, by forming a coexpression network module with TBR1, FEZF2, FOXG1, SATB2, and EMX1, plays a key role in development of the human neocortex and hippocampus projection neurons that are severely degenerated in AD brains [33]. All of these observations suggest that NeuroD6 acts as a key regulator of neuronal development, differentiation, and survival.

Previous studies identified LPPR4 and growth associated protein 43 (GAP43) as direct target genes for NeuroD6 by binding assay to E-boxes located in target gene promoters [24, 34]. Importantly, we found that the core set of 522 DEGs of RNA-Seq include LPPR as one of the downregulated genes in AD brains of both cohorts, and the study also identified GAP43 as a downregulated gene in AD brains of the UKY cohort (not shown). Although the precise biological role of NeuroD6 and its target genes in adult human brains remains unknown, the present observations suggest that downregulation of NeuroD6 might be detrimental for neuronal survival under stressful conditions caused by extensive accumulation of extracellular A*β* and intracellular NFT in AD.

In conclusion, the present study using bioinformatics data mining approach suggested that downregulation of NeuroD6 serves as a possible biomarker for diagnosis of AD by brain transcriptome profiling. Since the sample sizes we studied are apparently small, these findings warrant further validation in larger cohorts of AD patients and adequate controls performed in a blinded manner.

## Supplementary Material

Supplementary Figure 1: ROC analysis of microarray data GSE5281 by using NeuroD6 as a classifier. Transcriptome of LCM-captured frontal cortex neurons of age-matched AD patients and NC subjects was studied on a Human Genome U133 Plus 2.0 Array. The data normalized by MAS5 were transformed to Log2 values. ROC analysis was performed by importing the expression levels of NeuroD6 (panel a) or NeuroD1 (panel b) into SPSS in the setting of the group discrimination number 0 for AD and 1 for NC. The area under the ROC curve (AUC) is 0.893 for NeuroD6 and 0.474 for NeuroD1.
Supplementary Table 1: The core set of 522 DEGs in the frontal cortex of AD overlapping between two cohorts identified by RNA-Seq data analysis of SRA060752.
Supplementary Table 2: The set of 60 differentially spliced genes in the frontal cortex of AD identified by RNA-Seq data analysis of SRA060752.
Supplementary Table 3: The set of 215 DEGs in cortical neurons of the superior frontal gyrus of AD identified by microarray data analysis of GSE5281.
Supplementary Table 4: The set of 131 DEGs downregulated in the hippocampal CA1 region during progression of AD identified by microarray data analysis of GSE1297.
Supplementary Table 5: The set of 31 DEGs downregulated in the hippocampus of Japanese AD patients identified by microarray data analysis of GSE36980.

## Figures and Tables

**Table 1:** Top 20 DEGs in the frontal cortex of AD overlapping between two cohorts identified by RNA-Seq data analysis of SRA060752.

Entrez Gene ID	Gene symbol	Gene name	Chromosome locus	Fold change (AD versus NC: EMU)	*q*-value (EMU)	Fold change (AD versus NC: UKY)	*q*-value (UKY)
6863	TAC1	Tachykinin, precursor 1	chr7: 97361270–97369784	0.028013577	0.00232257	0.189297859	0.000638422
5121	PCP4	Purkinje cell protein 4	chr21: 41239346–41301322	0.060477403	0.00232257	0.263070248	0.000638422
793	CALB1	Calbindin 1, 28 kDa	chr8: 91070837–91095107	0.068756027	0.00232257	0.229478357	0.000638422
54112	GPR88	G protein-coupled receptor 88	chr1: 101002397–101008223	0.083735314	0.00232257	0.380695653	0.000638422
143162	FRMPD2	FERM and PDZ domain containing 2	chr10: 49364156–49482941	0.086014876	0.00232257	0.344904773	0.00248927
6588	SLN	Sarcolipin	chr11: 107578100–107582787	0.091647542	0.00398798	0.110806929	0.00396642
6750	SST	Somatostatin	chr3: 187386693–187388201	0.094938693	0.00232257	0.042406817	0.000638422
*63974 *	*NeuroD6 *	*Neurogenic differentiation 6 *	chr7: 31377079–31380538	0.095418314	0.00232257	0.159354316	0.000638422
3358	HTR2C	5-Hydroxytryptamine (serotonin) receptor 2C	chrX: 113818550–114144627	0.09625865	0.00232257	0.211803744	0.000638422
771	CA12	Carbonic anhydrase XII	chr15: 63615729–63674075	0.104187926	0.00232257	0.453231488	0.0218249

845	CASQ2	Calsequestrin 2 (cardiac muscle)	chr1: 116242519–116345204	3.491410523	0.00962874	5.322706439	0.000638422
23704	KCNE4	Potassium voltage-gated channel, Isk-related family, member 4	chr2: 223916861–223920355	4.436801472	0.00232257	4.699574041	0.000638422
6279	S100A8	S100 calcium binding protein A8	chr1: 153362507–153363664	5.018965399	0.00232257	2.142150734	0.0151369
871	SERPINH1	Serpin peptidase inhibitor, clade H (heat shock protein 47), member 1 (collagen binding protein 1)	chr11: 75273100–75283870	5.437863735	0.00232257	2.129345418	0.0101566
6275	S100A4	S100 calcium binding protein A4	chr1: 153516094–153518282	5.52659158	0.00232257	4.606560625	0.000638422
26266	SLC13A4	Solute carrier family 13 (sodium/sulfate symporters), member 4	chr7: 135365246–135412933	6.580968881	0.00232257	2.996233377	0.000638422
3303	HSPA1A	Heat shock 70 kDa protein 1A	chr6: 31777395–31785719	6.599057552	0.00232257	3.8880048	0.000638422
3304	HSPA1B	Heat shock 70 kDa protein 1B	chr6: 31795511–31798031	6.599057552	0.00232257	2.366327899	0.000638422
375061	FAM89A	Family with sequence similarity 89, member A	chr1: 231154703–231175995	8.186858725	0.00232257	2.253222839	0.00248927
100500849	MIR3916	MicroRNA 3916	chr1: 247342111–247374105	17.2291545	0.00762148	2.127294836	0.00287725

The core set of 522 DEGs in the frontal cortex of AD overlapping between EMU and UKY satisfying *q*-value (FDR-corrected *P* value) <0.05 and fold change greater than 2.0 or smaller than 0.5 were extracted by RNA-Seq data analysis of SRA060572. Top 10 downregulated and top 10 upregulated genes based on fold change in EMU are listed with Entrez Gene ID, gene symbol, gene name, chromosomal locus, fold change, and *q*-value. NeuroD6 is italicized. The complete list of 522 DEGs is shown in Supplementary Table  1.

**Table 2:** The set of 15 genes DEGs downregulated in cortical neurons of the superior frontal gyrus of AD identified by microarray data analysis of GSE5281 corresponding to RNA-Seq data analysis of SRA060752.

Entrez Gene ID	Gene symbol	Gene name	Fold change (AD versus NC)	*P* value
116	ADCYAP1	Adenylate cyclase activating polypeptide 1 (pituitary)	0.069636923	1.26039*E* − 07
6750	SST	Somatostatin	0.078547047	2.49899*E* − 05
10777	ARPP21	cAMP-regulated phosphoprotein, 21 kDa	0.1474134	4.52823*E* − 06
6511	SLC1A6	Solute carrier family 1 (high affinity aspartate/glutamate transporter), member 6	0.156759363	4.74541*E* − 05
728192	LINC00460	Long intergenic non-protein-coding RNA 460	0.171656016	8.49002*E* − 07
891	CCNB1	Cyclin B1	0.182817517	1.34014*E* − 05
523	ATP6V1A	ATPase, H+ transporting, lysosomal 70 kDa, V1 subunit A	0.190442377	8.45909*E* − 05
7991	TUSC3	Tumor suppressor candidate 3	0.200399391	3.74354*E* − 05
1741	DLG3	Discs, large homolog 3 (*Drosophila*)	0.222930685	8.59311*E* − 06
*63974 *	*NeuroD6 *	*Neurogenic differentiation 6 *	0.237572737	6.60728*E* − 05
3382	ICA1	Islet cell autoantigen 1, 69 kDa	0.249762845	2.41638*E* − 05
844	CASQ1	Calsequestrin 1 (fast-twitch, skeletal muscle)	0.271852315	3.03481*E* − 05
9577	BRE	Brain and reproductive organ-expressed (TNFRSF1A modulator)	0.306075086	9.92354*E* − 05
84900	RNFT2	Ring finger protein, transmembrane 2	0.306171729	4.52424*E* − 05
9515	STXBP5L	Syntaxin binding protein 5-like	0.360338998	8.58457*E* − 05

The set of 215 DEGs in LCM-captured frontal cortex neurons of AD satisfying *P* value <0.0001 by two-tailed *t*-test and fold change greater than 2 or smaller than 0.5 were extracted by microarray data analysis of GSE5281. Among them, the set of 15 genes corresponding to the core set of 522 DEGs identified by RNA-Seq data analysis of SRA060572 are listed with Entrez Gene ID, gene symbol, gene name, fold change, and *P* value. NeuroD6 is italicized. The complete set of 215 DEGs are shown in Supplementary Table  3.

**Table 3:** The set of 25 DEGs downregulated in the hippocampal CA1 region during progression of AD identified by microarray data analysis of GSE1297 corresponding to RNA-Seq data analysis of SRA060752.

Entrez Gene ID	Gene symbol	Gene name	Fold change (severe AD versus NC)	*P* value
57172	CAMK1G	Calcium/calmodulin-dependent protein kinase IG	0.119791216	0.00015044
7447	VSNL1	Visinin-like 1	0.176659556	0.003811085
10368	CACNG3	Calcium channel, voltage-dependent, gamma subunit 3	0.212906942	0.000645324
55711	FAR2	Fatty acyl CoA reductase 2	0.230090829	0.00066547
10769	PLK2	Polo-like kinase 2	0.264329482	0.001588852
9331	B4GALT6	UDP-Gal: betaGlcNAc beta 1,4-galactosyltransferase, polypeptide 6	0.284246871	0.005450676
55312	RFK	Riboflavin kinase	0.326351561	0.000250327
5274	SERPINI1	Serpin peptidase inhibitor, clade I (neuroserpin), member 1	0.337526915	0.006332563
9079	LDB2	LIM domain binding 2	0.340999225	0.003326867
1268	CNR1	Cannabinoid receptor 1 (brain)	0.344653589	0.008057005
5579	PRKCB	Protein kinase C, beta	0.345004889	0.002186833
63982	ANO3	Anoctamin 3	0.372084936	0.008600399
81831	NETO2	Neuropilin (NRP) and tolloid- (TLL-) like 2	0.393977566	0.000965658
440270	GOLGA8B	Golgin A8 family, member B	0.422538873	0.004711934
23236	PLCB1	Phospholipase C, beta 1 (phosphoinositide-specific)	0.43589904	0.001873881
27324	TOX3	TOX high mobility group box family member 3	0.450425735	0.002724595
6000	RGS7	Regulator of G-protein signaling 7	0.451971533	0.006045425
138046	RALYL	RALY RNA binding protein-like	0.453463038	0.001029166
5530	PPP3CA	Protein phosphatase 3, catalytic subunit, alpha isozyme	0.461727097	0.001385837
1020	CDK5	Cyclin-dependent kinase 5	0.464742579	0.00340998
3751	KCND2	Potassium voltage-gated channel, Shal-related subfamily, member 2	0.489787298	0.004105453
29114	TAGLN3	Transgelin 3	0.536481218	0.00215522
7534	YWHAZ	Tyrosine 3-monooxygenase/tryptophan 5-monooxygenase activation protein, zeta polypeptide	0.556167619	0.006101886
*63974 *	*NeuroD6 *	*Neurogenic differentiation 6 *	0.568549476	0.007199422
2744	GLS	Glutaminase	0.591660135	0.007897416

The set of 131 DEGs downregulated in the hippocampal CA1 region among incipient, moderate, and severe AD and NC groups by one-way ANOVA satisfying *P* value <0.01 and fold change of severe AD versus NC smaller than 0.6 were extracted by microarray data analysis of GSE1297. Among them, the set of 25 genes corresponding to the core set of 522 DEGs identified by RNA-Seq data analysis of SRA060572 are listed with Entrez Gene ID, gene symbol, gene name, fold change, and *P* value. NeuroD6 is italicized. The complete list of 131 DEGs are shown in Supplementary Table  4.

**Table 4:** The set of 12 DEGs downregulated in the hippocampus of Japanese AD patients identified by microarray data analysis of GSE36980 corresponding to RNA-Seq data analysis of SRA060752.

Entrez Gene ID	Gene symbol	Gene name	Fold change (AD versus non-AD)	*P* value
*63974 *	*NeuroD6 *	*Neurogenic differentiation 6 *	0.433171474	0.001616741
10368	CACNG3	Calcium channel, voltage-dependent, gamma subunit 3	0.496685808	0.004499281
5176	SERPINF1	Serpin peptidase inhibitor, clade F (alpha-2 antiplasmin, pigment epithelium derived factor), member 1	0.522226034	0.00019849
348980	HCN1	Hyperpolarization activated cyclic nucleotide-gated potassium channel 1	0.523810594	0.004508668
5774	PTPN3	Protein tyrosine phosphatase, nonreceptor type 3	0.54910964	0.001537752
8507	ENC1	Ectodermal-neural cortex (with BTB-like domain)	0.559652363	0.003288053
266722	HS6ST3	Heparan sulfate 6-O-sulfotransferase 3	0.560440342	0.001753288
2903	GRIN2A	Glutamate receptor, ionotropic, N-methyl D-aspartate 2A	0.581203611	0.002390123
51299	NRN1	Neuritin 1	0.590013553	0.001828335
125113	KRT222	Keratin 222 pseudogene	0.592296133	0.004941811
1428	CRYM	Crystallin, mu	0.594516424	0.002824762
221692	PHACTR1	Phosphatase and actin regulator 1	0.597310446	0.002464967

The set of 31 DEGs downregulated in the hippocampus of Japanese AD patients satisfying *P* value <0.005 by two-tailed *t*-test and fold change smaller than 0.6 were extracted by microarray data analysis of GSE36980. Among them, the set of 12 genes corresponding to the core set of 522 DEGs identified by RNA-Seq data analysis of SRA060572 are listed with Entrez Gene ID, gene symbol, gene name, fold change, and *P* value. NeuroD6 is italicized. The complete list of 31 DEGs are shown in Supplementary Table  5.
