# Supplementary material for: RNA-Seq Data Mining: Downregulation of NeuroD6 Serves as a Possible Biomarker for Alzheimer's Disease Brains
Source: Dis Markers. 2014 Dec 8;2014:123165. doi: 10.1155/2014/123165 (PMC4274867; doi:10.1155/2014/123165)
Supplement: Supplementary file 1 — Supplementary Figure 1: ROC analysis of microarray data GSE5281 by using NeuroD6 as a classifier. Transcriptome of LCM-captured frontal cortex neurons of age-matched AD patients and NC subjects was studied on a Human Genome U133 Plus 2.0 Array. The data normalized by MAS5 were transformed to Log2 values. ROC analysis was performed by importing the expression levels of NeuroD6 (panel a) or NeuroD1 (panel b) into SPSS in the setting of the group discrimination number 0 for AD and 1 for NC. The area under the ROC curve (AUC) is 0.893 for NeuroD6 and 0.474 for NeuroD1. Supplementary Table 1: The core set of 522 DEGs in the frontal cortex of AD overlapping between two cohorts identified by RNA-Seq data analysis of SRA060752. Supplementary Table 2: The set of 60 differentially spliced genes in the frontal cortex of AD identified by RNA-Seq data analysis of SRA060752. Supplementary Table 3: The set of 215 DEGs in cortical neurons of the superior frontal gyrus of AD identified by microarray data analysis of GSE5281. Supplementary Table 4: The set of 131 DEGs downregulated in the hippocampal CA1 region during progression of AD identified by microarray data analysis of GSE1297. Supplementary Table 5: The set of 31 DEGs downregulated in the hippocampus of Japanese AD patients identified by microarray data analysis of GSE36980. [file 123165.f1.pdf]

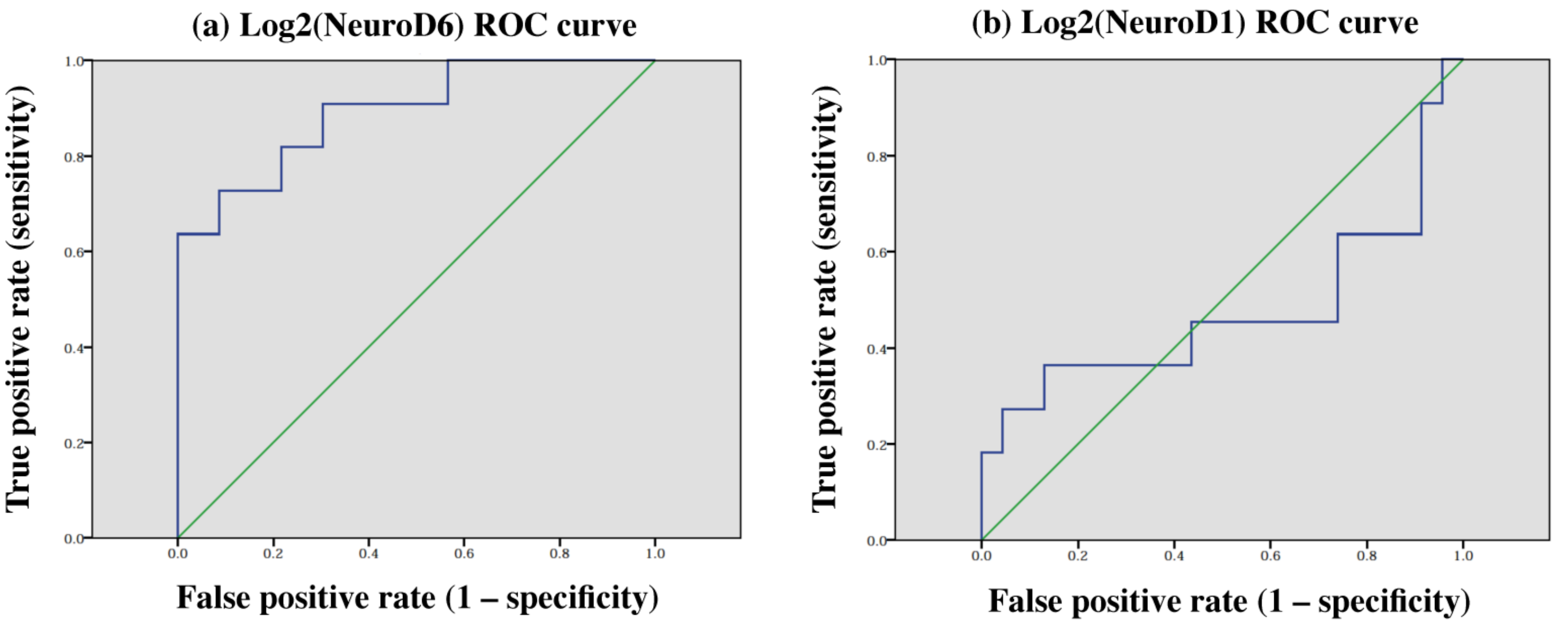

Supplementary Figure 1

Supplementary TABLE 1. The core set of 522 DEGs in the frontal cortex of AD overlapping between two cohorts identified by RNA-Seq data analysis of SRA060752

Entrez Gene ID	Gene Symbol	Gene Name	Chromosome Locus	Fold Change (AD vs NC: EMU)	q_Value (EMU)	Fold Change (AD vs NC: UKY)	q_Value (UKY)
6863	TAC1	tachykinin, precursor 1	chr7:97361270-97369784	0.028013577	0.00232257	0.189297859	0.000638422
5121	PCP4	Purkinje cell protein 4	chr21:41239346-41301322	0.060477403	0.00232257	0.263070248	0.000638422
793	CALB1	calbindin 1, 28kDa	chr8:91070837-91095107	0.068756027	0.00232257	0.229478357	0.000638422
54112	GPR88	G protein-coupled receptor 88	chr1:101002397-101008223	0.083735314	0.00232257	0.380695653	0.000638422
143162	FRMPD2	FERM and PDZ domain containing 2	chr10:49364156-49482941	0.086014876	0.00232257	0.344904773	0.00248927
6588	SLN	sarcolipin	chr11:107578100-107582787	0.091647542	0.00398798	0.110806929	0.00396642
6750	SST	somatostatin	chr3:187386693-187388201	0.094938693	0.00232257	0.042406817	0.000638422
63974	NEUROD6	neurogenic differentiation 6	chr7:31377079-31380538	0.095418314	0.00232257	0.159354316	0.000638422
3358	HTR2C	5-hydroxytryptamine (serotonin) receptor 2C	chrX:113818550-114144627	0.09625865	0.00232257	0.211803744	0.000638422
771	CA12	carbonic anhydrase XII	chr15:63615729-63674075	0.104187926	0.00232257	0.453231488	0.0218249
7432	VIP	vasoactive intestinal peptide	chr6:153053815-153080902	0.117494082	0.00232257	0.404586476	0.0139553
400120	SERTM1	serine-rich and transmembrane domain containing 1	chr13:37248048-37272564	0.130991037	0.00232257	0.151677606	0.000638422
63982	ANO3	anoctamin 3	chr11:26353677-26684908	0.137602635	0.00232257	0.269198075	0.000638422
5475	PPEF1	protein phosphatase, EF-hand calcium binding domain 1	chrX:18709044-18846034	0.145536205	0.00398798	0.148286586	0.000638422
728192	LINC00460	long intergenic non-protein coding RNA 460	chr13:107028910-107030142	0.147139857	0.0347863	0.181248379	0.00741018
10777	ARPP21	cyclic AMP-regulated phosphoprotein, 21 kD	chr3:35683848-35836031	0.149290058	0.00232257	0.422557624	0.000638422
7857	SCG2	secretogranin II (chromogranin C)	chr2:224461657-224476386	0.154096927	0.00232257	0.202705902	0.000638422
170261	ZCCHC12	zinc finger, CCHC domain containing 12	chrX:117957657-117960936	0.17100258	0.00232257	0.290566442	0.000638422
654790	PCP4L1	Purkinje cell protein 4 like 1	chr1:161228516-161255807	0.177964329	0.00232257	0.323897671	0.000638422
94122	SYTL5	synaptotagmin-like 5	chrX:37798907-37988225	0.181102705	0.00232257	0.326433268	0.000638422
24141	C20orf103	chromosome 20 open reading frame 103	chr20:9494940-9511408	0.188433843	0.00232257	0.393176773	0.000638422
4160	MC4R	melanocortin 4 receptor	chr18:58038563-58040001	0.198906179	0.0362874	0.402957614	0.0314924
129607	CMPK2	cytidine monophosphate (UMP-CMP) kinase 2, mitochondrial	chr2:6988450-7005936	0.199306408	0.00232257	0.44726135	0.00287725
728798	FRMPD2P1	FERM and PDZ domain containing 2 pseudogene 1	chr10:48844035-48862688	0.207716827	0.0131757	0.340732153	0.0243163
140767	NRSN1	neurensin 1	chr6:24126413-24147757	0.212544967	0.00232257	0.312550237	0.000638422
132204	SYNPR	synaptoporin	chr3:63263913-63602606	0.21334497	0.00232257	0.373013565	0.000638422
284424	MIR7-3HG	MIR7-3 host gene (non-protein coding)	chr19:4769025-4772807	0.214336581	0.00232257	0.273981427	0.000638422
83988	NCALD	neurocalcin delta	chr8:102698769-103137135	0.216218219	0.00232257	0.196063108	0.000638422
1428	CRYM	crystallin, mu	chr16:21269838-21329912	0.216835068	0.00232257	0.217688938	0.000638422
5122	PCSK1	proprotein convertase subtilisin/kexin type 1	chr5:95726039-95768985	0.21753056	0.00232257	0.142236142	0.000638422
1114	CHGB	chromogranin B (secretogranin 1)	chr20:5891973-5906005	0.220743062	0.00232257	0.173488267	0.000638422
157574	FBXO16	F-box protein 16	chr8:28285841-28347790	0.22144495	0.00232257	0.20520247	0.000638422
5999	RGS4	regulator of G-protein signaling 4	chr1:163038395-163046592	0.221819792	0.00232257	0.179382369	0.000638422
2571	GAD1	glutamate decarboxylase 1 (brain, 67kDa)	chr2:171673199-171717662	0.227504975	0.00232257	0.236188397	0.000638422
55359	STYK1	serine/threonine/tyrosine kinase 1	chr12:10769247-10826891	0.231383838	0.00539633	0.286252461	0.000638422
127733	UBXN10	UBX domain protein 10	chr1:20510733-20525453	0.231870309	0.00539633	0.269427684	0.000638422
115827	RAB3C	RAB3C, member RAS oncogene family	chr5:57878884-58155409	0.23314833	0.00398798	0.199877779	0.000638422
94160	ABCC12	ATP-binding cassette, sub-family C (CFTR/MRP), member 12	chr16:48116877-48180681	0.234178414	0.0452264	0.20178768	0.000638422
57687	VAT1L	vesicle amine transport protein 1 homolog (T. californica)-like	chr16:77822482-78014005	0.235758224	0.00232257	0.358841835	0.000638422
59350	RXFP1	relaxin/insulin-like family peptide receptor 1	chr4:159442853-159574534	0.23733553	0.00232257	0.264201397	0.000638422
219621	C10orf107	chromosome 10 open reading frame 107	chr10:63422718-63526501	0.243548331	0.0123074	0.405473631	0.0220269
90161	HS6ST2	heparan sulfate 6-O-sulfotransferase 2	chrX:131760037-132095423	0.244380318	0.00232257	0.426237668	0.00163713
389073	C2orf80	chromosome 2 open reading frame 80	chr2:209030070-209054969	0.245315449	0.00232257	0.344057119	0.000638422
54836	BSPRY	B-box and SPRY domain containing	chr9:116111805-116133513	0.248317871	0.0282007	0.484597881	0.049546
284485	RIIAD1	regulatory subunit of type II PKA R-subunit (RIIa) domain containing 1	chr1:151694012-151707982	0.252756483	0.0171483	0.281145722	0.0276375
54511	HMGCLL1	3-hydroxymethyl-3-methylglutaryl-Coenzyme A lyase-like 1	chr6:55299141-55444012	0.253101857	0.00232257	0.333063693	0.000638422
4905	NSF	N-ethylmaleimide-sensitive factor	chr17:44668034-44834828	0.255979468	0.00232257	0.310826196	0.000638422
27077	B9D1	B9 protein domain 1	chr17:19240358-19266233	0.258651238	0.00232257	0.352774944	0.00116486
84871	AGBL4	ATP/GTP binding protein-like 4	chr1:48998526-50489626	0.259211207	0.0330887	0.242894209	0.0190564
195814	SDR16C5	short chain dehydrogenase/reductase family 16C, member 5	chr8:57212385-57233241	0.259570799	0.00654308	0.285123726	0.000638422
25977	NECAP1	NECAP endocytosis associated 1	chr12:8234806-8250373	0.259781392	0.00232257	0.383338286	0.000638422
10962	MLLT11	myeloid/lymphoid or mixed-lineage leukemia (trithorax homolog, Drosophila); translocated to, 11	chr1:151032150-151040973	0.260977982	0.00232257	0.33308678	0.000638422
56884	FSTL5	follistatin-like 5	chr4:162305048-163085186	0.261686245	0.00232257	0.36597576	0.000638422
6860	SYT4	synaptotagmin IV	chr18:40847856-40857615	0.26263116	0.00232257	0.3177381	0.000638422
2566	GABRG2	gamma-aminobutyric acid (GABA) A receptor, gamma 2	chr5:161488463-161582794	0.263154141	0.00232257	0.251440683	0.000638422
6866	TAC3	tachykinin 3	chr12:57403780-57410344	0.265757074	0.00232257	0.392555896	0.0059412
5874	RAB27B	RAB27B, member RAS oncogene family	chr18:52385085-52562825	0.268447151	0.0104723	0.20489121	0.000638422
2554	GABRA1	gamma-aminobutyric acid (GABA) A receptor, alpha 1	chr5:161274196-161326965	0.269179417	0.00232257	0.246027246	0.000638422
2742	GLRA2	glycine receptor, alpha 2	chrX:14547419-14749935	0.269205539	0.00762148	0.313038064	0.000638422
5744	PTHLH	parathyroid hormone-like hormone	chr12:28111016-28124916	0.269211137	0.0485449	0.387073649	0.0172531
118427	OLFM3	olfactomedin 3	chr1:102263685-102462790	0.270484907	0.00232257	0.215399977	0.000638422
4345	CD200	CD200 molecule	chr3:112051517-112081658	0.271344957	0.00232257	0.350824158	0.000638422
84830	C6orf105	chromosome 6 open reading frame 105	chr6:11712570-11788090	0.273493794	0.0427273	0.165834951	0.000638422
3251	HPRT1	hypoxanthine phosphoribosyltransferase 1	chrX:133594174-133634698	0.275123256	0.00232257	0.295109036	0.000638422
8382	NME5	non-metastatic cells 5, protein expressed in (nucleoside-diphosphate kinase)	chr5:137450860-137475132	0.276133916	0.00232257	0.400079885	0.000638422
10486	CAP2	CAP, adenylate cyclase-associated protein, 2 (yeast)	chr6:17393735-17558023	0.277148288	0.00232257	0.244322731	0.000638422
627	BDNF	brain-derived neurotrophic factor	chr11:27528398-27743605	0.277782958	0.0358741	0.154546191	0.0337699
131034	CPNE4	copine IV	chr3:131252387-131756571	0.278019889	0.00232257	0.310105281	0.000638422
5058	PAK1	p21 protein (Cdc42/Rac)-activated kinase 1	chr11:77032592-77185108	0.278029525	0.00232257	0.229779182	0.000638422
5801	PTPRR	protein tyrosine phosphatase, receptor type, R	chr12:71031852-71314584	0.27804687	0.00232257	0.326512471	0.000638422
1268	CNR1	cannabinoid receptor 1 (brain)	chr6:88849584-88875767	0.278820782	0.00232257	0.428887151	0.000638422
407738	FAM19A1	family with sequence similarity 19 (chemokine (C-C motif)-like), member A1	chr3:68053453-68594771	0.280019655	0.00232257	0.282519039	0.000638422
285987	DLX6-AS1	DLX6 antisense RNA 1	chr7:96584450-96643377	0.280023537	0.00232257	0.320309835	0.000638422
1812	DRD1	dopamine receptor D1	chr5:174866960-174871939	0.2803673	0.00232257	0.497776491	0.0077184
6447	SCG5	secretogranin V (7B2 protein)	chr15:32933869-32989298	0.280466429	0.00232257	0.355973068	0.000638422
114569	MAL2	mal, T-cell differentiation protein 2	chr8:120220284-120257917	0.28067841	0.00232257	0.262436447	0.000638422
25827	FBXL2	F-box and leucine-rich repeat protein 2	chr3:33318933-33429075	0.280802951	0.00232257	0.335647779	0.000638422
80210	ARMC9	armadillo repeat containing 9	chr2:232063341-232220612	0.283704315	0.00398798	0.488520158	0.014683
7447	VSNL1	visinin-like 1	chr2:17721806-17837706	0.284109701	0.0270075	0.277344304	0.000638422
339524	LINC01140	long intergenic non-protein coding RNA 1140	chr1:87595447-87634886	0.284701105	0.00232257	0.275119442	0.000638422
127700	OSCP1	chromosome 1 open reading frame 102	chr1:36877383-36916086	0.286637645	0.00232257	0.438719276	0.00711203
9890	LPPR4	lipid phosphate phosphatase-related protein type 4	chr1:99729847-99775138	0.288377398	0.00232257	0.355521817	0.000638422
5650	KLK7	kallikrein-related peptidase 7	chr19:51479725-51487320	0.28890959	0.00232257	0.322927006	0.000638422
4190	MDH1	malate dehydrogenase 1, NAD (soluble)	chr2:63348534-63834336	0.289916631	0.00865834	0.308629955	0.000638422
2558	GABRA5	gamma-aminobutyric acid (GABA) A receptor, alpha 5	chr15:27111865-27194357	0.290483878	0.00232257	0.324227867	0.000638422
6511	SLC1A6	solute carrier family 1 (high affinity aspartate/glutamate transporter), member 6	chr19:15060845-15090560	0.295440599	0.00654308	0.278480845	0.000638422
2257	FGF12	fibroblast growth factor 12	chr3:191855146-192445388	0.296053538	0.00232257	0.293861854	0.000638422
55008	HERC6	hect domain and RLD 6	chr4:89299890-89364273	0.296836416	0.00232257	0.49769369	0.00326775
2572	GAD2	glutamate decarboxylase 2 (pancreatic islets and brain, 65kDa)	chr10:26504813-26593491	0.297396588	0.00232257	0.177175374	0.000638422
55711	FAR2	fatty acyl CoA reductase 2	chr12:29301861-29534143	0.297668817	0.00865834	0.352562271	0.0257176
2556	GABRA3	gamma-aminobutyric acid (GABA) A receptor, alpha 3	chrX:151334703-151619869	0.298338072	0.00232257	0.297976405	0.000638422
8001	GLRA3	glycine receptor, alpha 3	chr4:175557690-175750638	0.298418731	0.00865834	0.292445562	0.000638422
113802	HENMT1	HEN1 methyltransferase homolog 1 (Arabidopsis)	chr1:109190909-109204148	0.298511828	0.00232257	0.488696271	0.0220269
6616	SNAP25	synaptosomal-associated protein, 25kDa	chr20:10002241-10288066	0.299951261	0.020736	0.266559591	0.000638422
6252	RTN1	reticulon 1	chr14:60062628-60337557	0.302267967	0.00232257	0.339010062	0.000638422

6092	ROBO2	roundabout, axon guidance receptor, homolog 2 (Drosophila)	chr3:77089293-77699114	0.30322277	0.00232257	0.233337486	0.000638422
124925	SEZ6	seizure related 6 homolog (mouse)	chr17:27281946-27333081	0.306188075	0.00232257	0.358540998	0.000638422
6014	RIT2	Ras-like without CAAX 2	chr18:40323191-40695707	0.306549084	0.00232257	0.468356827	0.00825241
1174	AP1S1	adaptor-related protein complex 1, sigma 1 subunit	chr7:100797685-100804557	0.307210621	0.00232257	0.403485854	0.000638422
84100	ARL6	ADP-ribosylation factor-like 6	chr3:97482034-97537311	0.3075302	0.00232257	0.439178703	0.013009
51617	HMP19	HMP19 protein	chr5:173472723-173536230	0.307749837	0.00232257	0.300057314	0.000638422
5274	SERPINI1	serpin peptidase inhibitor, clade I (neuroserpin), member 1	chr3:167453431-167543357	0.308371213	0.00232257	0.367959784	0.000638422
122402	TDRD9	tudor domain containing 9	chr14:104394816-104519006	0.309585541	0.00232257	0.379625813	0.00961203
4753	NELL2	NEL-like 2 (chicken)	chr12:44902024-45315614	0.310677572	0.00232257	0.299857716	0.000638422
10056	FARSB	phenylalanyl-tRNA synthetase, beta subunit	chr2:223436161-223520827	0.311112874	0.00232257	0.475421812	0.000638422
57512	GPR158	G protein-coupled receptor 158	chr10:25447000-25891164	0.311998284	0.00232257	0.286836396	0.000638422
3739	KCNA4	potassium voltage-gated channel, shaker-related subfamily, member 4	chr11:30028707-30038675	0.312043702	0.00398798	0.451231597	0.00625285
6622	SNCA	synuclein, alpha (non A4 component of amyloid precursor)	chr4:90645249-90759447	0.312914409	0.00398798	0.341708964	0.000638422
7277	TUBA4A	tubulin, alpha 4a	chr2:220110191-220136910	0.31587376	0.00962874	0.35502684	0.00116486
221692	PHACTR1	phosphatase and actin regulator 1	chr6:12717036-13296059	0.316226461	0.00232257	0.408936856	0.0428614
79589	RNF128	ring finger protein 128	chrX:105937067-106040246	0.316555419	0.039908	0.374407178	0.000638422
29118	DDX25	DEAD (Asp-Glu-Ala-Asp) box polypeptide 25	chr11:125773275-125799083	0.319060076	0.00232257	0.430555165	0.000638422
10368	CACNG3	calcium channel, voltage-dependent, gamma subunit 3	chr16:24266873-24373738	0.31952927	0.00232257	0.275003141	0.000638422
29089	UBE2T	ubiquitin-conjugating enzyme E2T (putative)	chr1:202300784-202311094	0.319653324	0.00232257	0.410606964	0.0059412
1006	CDH8	cadherin 8, type 2	chr16:61681155-62070739	0.32038311	0.00232257	0.267709455	0.000638422
613212	CTXN3	cortexin 3	chr5:126984712-126994322	0.321003292	0.00232257	0.265880523	0.000638422
84280	BTBD10	BTB (POZ) domain containing 10	chr11:13409286-13484838	0.321136822	0.00232257	0.454700974	0.000638422
55617	TASP1	taspase, threonine aspartase, 1	chr20:13370035-13619583	0.321243685	0.00232257	0.351686046	0.000638422
112609	MRAP2	melanocortin 2 receptor accessory protein 2	chr6:84730637-84800606	0.321791919	0.00232257	0.287680632	0.000638422
340542	BEX5	brain expressed, X-linked 5	chrX:101408678-101410986	0.322938198	0.00232257	0.253676187	0.000638422
2743	GLRB	glycine receptor, beta	chr4:157997276-158093242	0.323101645	0.00232257	0.428055569	0.000638422
5110	PCMT1	protein-L-isoaspartate (D-aspartate) O-methyltransferase	chr6:150070830-150132557	0.323336885	0.00232257	0.470067551	0.000638422
23543	RBFOX2	RNA binding protein, fox-1 homolog (C. elegans) 2	chr22:36134780-36424615	0.325653582	0.0104723	0.470012164	0.000638422
4085	MAD2L1	MAD2 mitotic arrest deficient-like 1 (yeast)	chr4:120979649-120988058	0.326213863	0.00232257	0.427092361	0.00287725
201229	C17orf108	hypothetical protein LOC201229	chr17:26205339-26318758	0.326358609	0.00232257	0.36152066	0.000638422
2805	GOT1	glutamic-oxaloacetic transaminase 1, soluble (aspartate aminotransferase 1)	chr10:101156626-101190530	0.326614331	0.00232257	0.296014551	0.000638422
125113	KRT222	keratin 222 pseudogene	chr17:38810627-38821440	0.327221625	0.00232257	0.314975168	0.000638422
84649	DGAT2	diacylglycerol O-acyltransferase homolog 2 (mouse)	chr11:75479777-75512805	0.327641498	0.0148125	0.401065563	0.00207151
4852	NPY	neuropeptide Y	chr7:24323806-24331484	0.328749362	0.00232257	0.264887193	0.000638422
5019	OXCT1	3-oxoacid CoA transferase 1	chr5:41730164-41870791	0.32905029	0.00232257	0.464400039	0.000638422
7534	YWHAZ	tyrosine 3-monooxygenase/tryptophan 5-monooxygenase activation protein, zeta polypeptide	chr8:101930803-101965623	0.329817533	0.00398798	0.471163602	0.00396642
116	ADCYAP1	adenylate cyclase activating polypeptide 1 (pituitary)	chr18:902790-912982	0.330850187	0.00232257	0.092915301	0.000638422
345079	ANKRD56	ankyrin repeat domain 56	chr4:77814750-77819319	0.331136971	0.020736	0.184642939	0.000638422
5800	PTPRO	protein tyrosine phosphatase, receptor type, O	chr12:15475190-15751328	0.331469952	0.00232257	0.338047996	0.000638422
29114	TAGLN3	transgelin 3	chr3:111717585-111732735	0.331690593	0.00398798	0.310727105	0.000638422
81533	ITFG1	integrin alpha FG-GAP repeat containing 1	chr16:47188295-47735545	0.332733724	0.00232257	0.418709048	0.00287725
4745	NELL1	NEL-like 1 (chicken)	chr11:20688967-21599776	0.332964437	0.00232257	0.205094399	0.000638422
55531	ELMOD1	ELMO/CED-12 domain containing 1	chr11:107461816-107537820	0.333315428	0.00232257	0.334725415	0.000638422
57569	ARHGAP20	Rho GTPase activating protein 20	chr11:110447764-110583451	0.333565041	0.00232257	0.385515294	0.000638422
1123	CHN1	chimerin (chimaerin) 1	chr2:175664041-175870107	0.335113102	0.0234694	0.393501216	0.00116486
401190	RGS7BP	regulator of G-protein signaling 7 binding protein	chr5:63801828-63910494	0.336682351	0.00232257	0.377034906	0.000638422
2983	GUCY1B3	guanylate cyclase 1, soluble, beta 3	chr4:156680125-156728796	0.337189145	0.00232257	0.376173467	0.000638422
10231	RCAN2	regulator of calcineurin 2	chr6:46188438-46459695	0.339336847	0.00232257	0.333923608	0.000638422
51559	NT5DC3	5'-nucleotidase domain containing 3	chr12:104166080-104235003	0.339428592	0.00232257	0.376562176	0.000638422
28512	NKIRAS1	NFKB inhibitor interacting Ras-like 1	chr3:23933571-23958537	0.339781686	0.00232257	0.471454353	0.00116486
283385	MORN3	MORN repeat containing 3	chr12:122086455-122110517	0.340170514	0.0492625	0.390266085	0.00532481
63908	NAPB	N-ethylmaleimide-sensitive factor attachment protein, beta	chr20:23355115-23402156	0.340555066	0.00232257	0.267057073	0.000638422
51382	ATP6V1D	ATPase, H+ transporting, lysosomal 34kDa, V1 subunit D	chr14:67708020-67826720	0.341254506	0.00539633	0.470566329	0.0406304
9201	DCLK1	doublecortin-like kinase 1	chr13:35516423-36705546	0.341469825	0.0131757	0.358339752	0.000638422
84707	BEX2	brain expressed X-linked 2	chrX:102564273-102565974	0.342881031	0.00232257	0.400832113	0.000638422
81849	ST6GALNAC5	ST6 (alpha-N-acetyl-neuraminyl-2,3-beta-galactosyl-1,3)-N-acetylgalactosaminide alpha-2,6-sialyltransferase 5	chr1:77332990-77537810	0.343604301	0.00762148	0.276377102	0.000638422
3360	HTR4	5-hydroxytryptamine (serotonin) receptor 4	chr5:147825370-148042212	0.34393313	0.00865834	0.387986941	0.00248927
9737	GPRASP1	G protein-coupled receptor associated sorting protein 1	chrX:101854095-101972669	0.345213311	0.0131757	0.367901127	0.000638422
5100	PCDH8	protocadherin 8	chr13:53418108-53422775	0.345371275	0.00539633	0.234267707	0.000638422
51807	TUBA8	tubulin, alpha 8	chr22:18593452-18614498	0.345579609	0.00232257	0.328398627	0.000638422
7991	TUSC3	tumor suppressor candidate 3	chr8:15397525-15624158	0.345785673	0.00232257	0.408732821	0.000638422
5136	PDE1A	phosphodiesterase 1A, calmodulin-dependent	chr2:183004766-183387465	0.347298961	0.00232257	0.394088075	0.000638422
29789	OLA1	Obg-like ATPase 1	chr2:174937174-175113367	0.347474738	0.00232257	0.454515059	0.000638422
29887	SNX10	sorting nexin 10	chr7:26331514-26413949	0.347534956	0.00232257	0.295123355	0.000638422
56341	PRMT8	protein arginine methyltransferase 8	chr12:3592894-3703140	0.348328398	0.00865834	0.173166288	0.000638422
55800	SCN3B	sodium channel, voltage-gated, type III, beta	chr11:123499814-123525315	0.348335641	0.00232257	0.284346115	0.000638422
5579	PRKCB	protein kinase C, beta	chr16:23847299-24231932	0.348482956	0.0257447	0.381908782	0.000638422
23057	NMNAT2	nicotinamide nucleotide adenylyltransferase 2	chr1:183217025-183387634	0.349000256	0.00232257	0.227246503	0.000638422
56954	NIT2	nitrilase family, member 2	chr3:100053559-100082147	0.350481452	0.00762148	0.447341962	0.0059412
5774	PTPN3	protein tyrosine phosphatase, non-receptor type 3	chr9:112137772-112260593	0.350668562	0.00232257	0.24653596	0.000638422
9079	LDB2	LIM domain binding 2	chr4:16503124-16932620	0.352108022	0.00232257	0.363080159	0.000638422
28969	BZW2	basic leucine zipper and W2 domains 2	chr7:16685758-16746148	0.352159279	0.00232257	0.468691326	0.00207151
22895	RPH3A	rabphilin 3A homolog (mouse)	chr12:113013741-113336688	0.352208102	0.00232257	0.169148232	0.000638422
529	ATP6V1E1	ATPase, H+ transporting, lysosomal 31kDa, V1 subunit E1	chr22:18074902-18111588	0.353134578	0.00232257	0.422956149	0.000638422
1607	DGKB	diacylglycerol kinase, beta 90kDa	chr7:14184458-15014651	0.35355094	0.00232257	0.267171866	0.000638422
2258	FGF13	fibroblast growth factor 13	chrX:137713733-138287185	0.353568095	0.00232257	0.299685253	0.000638422
11249	NXPH2	neurexophilin 2	chr2:139426726-139537811	0.353597505	0.0341672	0.25863331	0.000638422
4033	LRMP	lymphoid-restricted membrane protein	chr12:25205180-25348094	0.354530094	0.0387574	0.479332943	0.0101566
4674	NAP1L2	nucleosome assembly protein 1-like 2	chrX:72432136-72434710	0.355149904	0.00232257	0.321617987	0.000638422
401097	C3orf80	chromosome 3 open reading frame 80	chr3:159933976-159948942	0.355240999	0.00654308	0.20766788	0.000638422
9315	C5orf13	chromosome 5 open reading frame 13	chr5:110559913-111312628	0.355881785	0.00539633	0.429178586	0.0344432
486	FXYD2	FXYD domain containing ion transport regulator 2	chr11:117690789-117748201	0.356101397	0.00232257	0.358573307	0.000638422
53826	FXYD6	FXYD domain containing ion transport regulator 6	chr11:117690789-117748201	0.356101397	0.00232257	0.358573307	0.000638422
100533181	FXYD6-FXYD2	FXYD6-FXYD2 readthrough	chr11:117690789-117748201	0.356101397	0.00232257	0.358573307	0.000638422
1020	CDK5	cyclin-dependent kinase 5	chr7:150750217-150755052	0.356701701	0.00232257	0.406864412	0.000638422
9840	TESPA1	thymocyte expressed, positive selection associated 1	chr12:55341800-55378456	0.357285681	0.00232257	0.256937612	0.000638422
1007	CDH9	cadherin 9, type 2 (T1-cadherin)	chr5:26879337-27038689	0.358128689	0.00232257	0.322383542	0.000638422
526	ATP6V1B2	ATPase, H+ transporting, lysosomal 56/58kDa, V1 subunit B2	chr8:20054703-20079207	0.3594941	0.00232257	0.310171922	0.000638422
1958	EGR1	early growth response 1	chr5:137801180-137805004	0.36016253	0.00232257	0.378745329	0.000638422
51062	ATL1	atlastin GTPase 1	chr14:50999799-51135221	0.360684667	0.00232257	0.445034782	0.00207151
57282	SLC4A10	solute carrier family 4, sodium bicarbonate transporter, member 10	chr2:162480844-162841786	0.361084901	0.00232257	0.307997383	0.000638422
117154	DACH2	dachshund homolog 2 (Drosophila)	chrX:85403454-86087605	0.363606526	0.0123074	0.317256142	0.000638422
2835	GPR12	G protein-coupled receptor 12	chr13:27326863-27334929	0.364436661	0.00232257	0.356340903	0.000638422
1960	EGR3	early growth response 3	chr8:22545173-22550815	0.364727276	0.00232257	0.391233008	0.000638422
54033	RBM11	RNA binding motif protein 11	chr21:15588439-15600843	0.365005473	0.00539633	0.498232142	0.00825241
286002	SLC26A4-AS1	SLC26A4 antisense RNA 1	chr7:107296960-107358363	0.365045955	0.0477817	0.299261792	0.000638422
2259	FGF14	fibroblast growth factor 14	chr13:102104965-103054124	0.365083912	0.00232257	0.372597526	0.00326775
1780	DYNC1I1	dynein, cytoplasmic 1, intermediate chain 1	chr7:95401817-95727743	0.366508865	0.00232257	0.340595198	0.000638422
10769	PLK2	polo-like kinase 2 (Drosophila)	chr5:57749809-57755913	0.366640992	0.00232257	0.370016113	0.000638422
25849	PARM1	DKFZP564O0823 protein	chr4:75858284-75975429	0.366984237	0.00232257	0.273453987	0.000638422
6326	SCN2A	sodium channel, voltage-gated, type II, alpha subunit	chr2:166095911-166249080	0.368041409	0.00232257	0.314717651	0.000638422

10923	SUB1	SUB1 homolog (S. cerevisiae)	chr5:32585604-32604185	0.368838204	0.00232257	0.478768453	0.000638422
9615	GDA	guanine deaminase	chr9:74729552-74867140	0.368866327	0.00398798	0.209791772	0.000638422
79772	MCTP1	multiple C2 domains, transmembrane 1	chr5:93954390-94620284	0.368950711	0.00232257	0.352212984	0.000638422
55859	BEX1	brain expressed, X-linked 1	chrX:102317574-102319168	0.368978843	0.00232257	0.34393313	0.000638422
7070	THY1	Thy-1 cell surface antigen	chr11:119225922-119599435	0.369093951	0.00232257	0.324648399	0.00361782
254887	ZDHHC23	zinc finger, DHHC-type containing 23	chr3:113666747-113682214	0.369716158	0.00762148	0.278793726	0.000638422
140689	CBLN4	cerebellin 4 precursor	chr20:54571586-54580585	0.37028551	0.00654308	0.201259669	0.000638422
1996	ELAVL4	ELAV (embryonic lethal, abnormal vision, Drosophila)-like 4 (Hu antigen D)	chr1:50513685-50669481	0.373070451	0.00398798	0.356387836	0.000638422
81553	FAM49A	family with sequence similarity 49, member A	chr2:16730723-16847134	0.373168729	0.00232257	0.430355258	0.000638422
57552	NCEH1	arylacetamide deacetylase-like 1	chr3:172348325-172429101	0.373261858	0.00232257	0.435864014	0.000638422
23322	RPGRIP1L	RPGRIP1-like	chr16:53633047-53737771	0.373347248	0.00232257	0.43548653	0.00116486
5066	PAM	peptidylglycine alpha-amidating monooxygenase	chr5:102201522-102366808	0.374474659	0.00232257	0.448313547	0.000638422
7334	UBE2N	ubiquitin-conjugating enzyme E2N (UBC13 homolog, yeast)	chr12:93797227-93836026	0.374583693	0.00232257	0.492016976	0.000638422
22920	KIFAP3	kinesin-associated protein 3	chr1:169890469-170043894	0.375235961	0.00232257	0.437262036	0.000638422
64112	MOAP1	modulator of apoptosis 1	chr14:93648540-93651249	0.376303862	0.00232257	0.353636722	0.000638422
10687	PNMA2	paraneoplastic antigen MA2	chr8:26362192-26371483	0.376836339	0.00654308	0.351725052	0.000638422
90523	MLIP	muscular LMNA-interacting protein	chr6:53883713-54132612	0.377670499	0.0215169	0.19376367	0.000638422
51299	NRN1	neuritin 1	chr6:5998225-6007788	0.378225883	0.00232257	0.260156201	0.000638422
7345	UCHL1	ubiquitin carboxyl-terminal esterase L1 (ubiquitin thiolesterase)	chr4:41258897-41270446	0.378695452	0.0104723	0.332784467	0.000638422
5950	RBP4	retinol binding protein 4, plasma	chr10:95351592-95361206	0.379168232	0.00762148	0.291716723	0.000638422
57692	MAGEE1	melanoma antigen family E, 1	chrX:75648045-75651746	0.379223428	0.00232257	0.327042492	0.000638422
9215	LARGE	like-glycosyltransferase	chr22:33668490-34319757	0.37926023	0.00232257	0.424998602	0.000638422
144423	GLT1D1	glycosyltransferase 1 domain containing 1	chr12:129337983-129469741	0.379502159	0.0415504	0.3290138	0.000638422
54704	PDP1	pyruvate dehyrogenase phosphatase catalytic subunit 1	chr8:94929082-94938298	0.37965476	0.00232257	0.339207506	0.000638422
7259	TSPYL1	TSPY-like 1	chr6:116596021-116766108	0.380089216	0.00232257	0.378073857	0.000638422
121268	RHEBL1	Ras homolog enriched in brain like 1	chr12:49458459-49463775	0.381273984	0.016349	0.273455882	0.000638422
4168	MCF2	MCF.2 cell line derived transforming sequence	chrX:138663792-138790381	0.381459025	0.00232257	0.400593245	0.000638422
3775	KCNK1	potassium channel, subfamily K, member 1	chr1:233749749-233808365	0.381824081	0.00232257	0.481403989	0.000638422
100507043	TUNAR	TCL1 upstream neural differentiation-associated RNA	chr14:96343108-96391908	0.382218629	0.00762148	0.296587562	0.000638422
9899	SV2B	synaptic vesicle glycoprotein 2B	chr15:91643087-91845012	0.382385574	0.00232257	0.305689732	0.000638422
9317	PTER	phosphotriesterase related	chr10:16478966-16564004	0.382854999	0.00232257	0.231032865	0.000638422
6000	RGS7	regulator of G-protein signaling 7	chr1:240938814-241520754	0.383232017	0.01139	0.24325477	0.000638422
58527	C6orf115	chromosome 6 open reading frame 115	chr6:139349818-139364490	0.383580158	0.00232257	0.457368845	0.00361782
644150	WIPF3	WAS/WASL interacting protein family, member 3	chr7:29846169-29956682	0.384194827	0.00232257	0.36214014	0.000638422
9185	REPS2	RALBP1 associated Eps domain containing 2	chrX:16964813-17171412	0.384349314	0.00232257	0.454855436	0.000638422
55228	PNMAL1	PNMA-like 1	chr19:46969678-46974820	0.384373292	0.00232257	0.399711227	0.000638422
6857	SYT1	synaptotagmin I	chr12:79257772-79849213	0.384946538	0.0282007	0.234592696	0.000638422
9369	NRXN3	neurexin 3	chr14:78870092-80331069	0.385109335	0.00654308	0.340734515	0.000638422
84708	LNX1	ligand of numb-protein X 1	chr4:54243819-54457724	0.385408421	0.0305778	0.456934728	0.0109649
6990	DYNLT3	dynein, light chain, Tctex-type 3	chrX:37698088-37706889	0.38561685	0.00232257	0.427581103	0.000638422
9162	DGKI	diacylglycerol kinase, iota	chr7:137064445-137531609	0.386272265	0.00232257	0.441819806	0.000638422
1047	CLGN	calmegin	chr4:141309606-141348815	0.387586439	0.0131757	0.315471154	0.000638422
6547	SLC8A3	solute carrier family 8 (sodium/calcium exchanger), member 3	chr14:70510932-70656160	0.388635609	0.01139	0.417732125	0.00287725
2898	GRIK2	glutamate receptor, ionotropic, kainate 2	chr6:101830609-102517958	0.388735293	0.00232257	0.432955304	0.000638422
9577	BRE	brain and reproductive organ-expressed (TNFRSF1A modulator)	chr2:28004265-28561767	0.388910475	0.0437248	0.410262729	0.000638422
5217	PFN2	profilin 2	chr3:149682687-149688959	0.38901023	0.00865834	0.428316749	0.000638422
528	ATP6V1C1	ATPase, H+ transporting, lysosomal 42kDa, V1 subunit C1	chr8:104033247-104085285	0.3896336	0.00232257	0.453655797	0.000638422
100507421	TMEM178B	transmembrane protein 178B	chr7:140774031-141180181	0.390117332	0.00232257	0.420148139	0.000638422
5590	PRKCZ	protein kinase C, zeta	chr1:1981908-2139172	0.390864374	0.040699	0.496095577	0.0167892
6330	SCN4B	sodium channel, voltage-gated, type IV, beta	chr11:118004014-118023630	0.39096734	0.00232257	0.410698049	0.000638422
534	ATP6V1G2	ATPase, H+ transporting, lysosomal 13kDa, V1 subunit G2	chr6:31496738-31514625	0.391561276	0.00232257	0.417199693	0.000638422
7919	DDX39B	DEAD (Asp-Glu-Ala-Asp) box polypeptide 39B	chr6:31496738-31514625	0.391561276	0.00232257	0.417199693	0.000638422
7385	UQCRC2	ubiquinol-cytochrome c reductase core protein II	chr16:21964608-21994795	0.391629134	0.00398798	0.478363759	0.000638422
151473	SLC16A14	solute carrier family 16, member 14 (monocarboxylic acid transporter 14)	chr2:230899689-230933715	0.39175674	0.00654308	0.471065637	0.000638422
56479	KCNQ5	potassium voltage-gated channel, KQT-like subfamily, member 5	chr6:73331570-73908845	0.392096318	0.00232257	0.307021161	0.000638422
6854	SYN2	synapsin II	chr3:12045861-12233532	0.392210483	0.00232257	0.299002614	0.000638422
55335	NIPSNAP3B	nipsnap homolog 3B (C. elegans)	chr9:107526058-107540271	0.3930705	0.00654308	0.474282979	0.00825241
2026	ENO2	enolase 2 (gamma, neuronal)	chr12:7023613-7032859	0.393285799	0.020736	0.284430878	0.000638422
55294	FBXW7	F-box and WD repeat domain containing 7	chr4:153242409-153456172	0.393367589	0.0155725	0.394019791	0.000638422
9114	ATP6V0D1	ATPase, H+ transporting, lysosomal 38kDa, V0 subunit d1	chr16:67471916-67515089	0.394145443	0.00232257	0.41172701	0.000638422
27018	NGFRAP1	nerve growth factor receptor (TNFRSF16) associated protein 1	chrX:102631267-102633001	0.394435142	0.00762148	0.492098833	0.000638422
22911	WDR47	WD repeat domain 47	chr1:109512837-109584850	0.395527525	0.00232257	0.498954442	0.000638422
5504	PPP1R2	protein phosphatase 1, regulatory (inhibitor) subunit 2	chr3:195241220-195270224	0.395911533	0.00232257	0.475003484	0.000638422
2274	FHL2	four and a half LIM domains 2	chr2:105977282-106055230	0.396963977	0.00865834	0.44043774	0.00207151
138046	RALYL	RALY RNA binding protein-like	chr8:85095452-85834449	0.397269516	0.00232257	0.368375751	0.000638422
80823	BHLHB9	basic helix-loop-helix domain containing, class B, 9	chrX:101975641-102008748	0.397346626	0.00232257	0.493855198	0.000638422
5230	PGK1	phosphoglycerate kinase 1	chrX:77359665-77395179	0.398234468	0.00398798	0.45266636	0.00116486
9892	SNAP91	synaptosomal-associated protein, 91kDa homolog (mouse)	chr6:84262596-84419127	0.398251031	0.00398798	0.369093951	0.000638422
284611	FAM102B	family with sequence similarity 102, member B	chr1:109102970-109181949	0.398894739	0.00398798	0.367641109	0.000638422
9671	WSCD2	WSC domain containing 2	chr12:108523130-108644395	0.399503487	0.00232257	0.491604491	0.00432944
55061	SUSD4	sushi domain containing 4	chr1:223394160-223537544	0.40068211	0.0286978	0.31634704	0.000638422
2917	GRM7	glutamate receptor, metabotropic 7	chr3:6902801-7783452	0.401026645	0.0378873	0.469019562	0.0225012
85019	C18orf45	chromosome 18 open reading frame 45	chr18:20875313-21017925	0.401285241	0.0131757	0.429914001	0.00465363
7416	VDAC1	voltage-dependent anion channel 1	chr5:133307565-133340824	0.402089899	0.00232257	0.417136078	0.000638422
7754	ZNF204P	zinc finger protein 204 pseudogene	chr6:27325528-27343192	0.402279465	0.00398798	0.410923003	0.000638422
5634	PRPS2	phosphoribosyl pyrophosphate synthetase 2	chrX:12809473-12842346	0.40255003	0.00232257	0.471323657	0.000638422
785	CACNB4	calcium channel, voltage-dependent, beta 4 subunit	chr2:152689276-152955593	0.402910135	0.00232257	0.343530477	0.000638422
5176	SERPINF1	serpin peptidase inhibitor, clade F (alpha-2 antiplasmin, pigment epithelium derived factor), member 1	chr17:1665258-1680859	0.403284538	0.0179767	0.338981865	0.000638422
127254	C1orf173	chromosome 1 open reading frame 173	chr1:75033794-75140078	0.403796413	0.00539633	0.303986677	0.000638422
51256	TBC1D7	TBC1 domain family, member 7	chr6:13305181-13328787	0.404011986	0.00539633	0.445133505	0.00287725
59353	TMEM35	transmembrane protein 35	chrX:100333835-100351355	0.40422207	0.00232257	0.31658614	0.000638422
57559	STAMBPL1	STAM binding protein-like 1	chr10:90640025-90683271	0.404390217	0.0179767	0.440138659	0.00163713
79719	AAGAB	alpha- and gamma-adaptin-binding protein p34	chr15:67491063-67819641	0.404564041	0.00232257	0.487360084	0.00741018
27065	D4S234E	DNA segment on chromosome 4 (unique) 234 expressed sequence	chr4:4387982-4543775	0.405580445	0.0187073	0.278903897	0.00499267
6638	SNRPN	small nuclear ribonucleoprotein polypeptide N	chr15:25068793-25223729	0.406089604	0.0253156	0.440639276	0.000638422
8926	SNURF	SNRPN upstream reading frame	chr15:25068793-25223729	0.406089604	0.0253156	0.440639276	0.000638422
54332	GDAP1	ganglioside-induced differentiation-associated protein 1	chr8:75233751-75279335	0.407476849	0.00398798	0.337018572	0.000638422
23285	KIAA1107	KIAA1107	chr1:92545861-92656269	0.40750227	0.00232257	0.436145074	0.000638422
284697	BTBD8	BTB (POZ) domain containing 8	chr1:92545861-92656269	0.40750227	0.00232257	0.436145074	0.000638422
283209	PGM2L1	phosphoglucomutase 2-like 1	chr11:74041360-74109502	0.408540214	0.00654308	0.251228144	0.000638422
10947	AP3M2	adaptor-related protein complex 3, mu 2 subunit	chr8:42010463-42028705	0.408571365	0.00232257	0.435254162	0.000638422
55847	CISD1	CDGSH iron sulfur domain 1	chr10:60028802-60052654	0.409663141	0.0496454	0.396386573	0.000638422
26034	IPCEF1	interaction protein for cytohesin exchange factors 1	chr6:154331635-154677900	0.410484599	0.00654308	0.287192504	0.000638422
266722	HS6ST3	heparan sulfate 6-O-sulfotransferase 3	chr13:96743092-97491812	0.411122433	0.00398798	0.287904052	0.000638422
114815	SORCS1	sortilin-related VPS10 domain containing receptor 1	chr10:108333420-108924466	0.411339066	0.00232257	0.424059909	0.000638422
523	ATP6V1A	ATPase, H+ transporting, lysosomal 70kDa, V1 subunit A	chr3:113465865-113530905	0.412503996	0.00762148	0.35181283	0.000638422
54331	GNG2	guanine nucleotide binding protein (G protein), gamma 2	chr14:52314019-52436518	0.413013258	0.00232257	0.374822639	0.000638422
1993	ELAVL2	ELAV (embryonic lethal, abnormal vision, Drosophila)-like 2 (Hu antigen B)	chr9:23690098-23826457	0.414004972	0.00232257	0.3344958	0.000638422
51606	ATP6V1H	ATPase, H+ transporting, lysosomal 50/57kDa, V1 subunit H	chr8:54628100-54755850	0.41425758	0.00232257	0.426211079	0.000638422
92691	TMEM169	transmembrane protein 169	chr2:216946588-216967506	0.414335115	0.043185	0.372060722	0.000638422
57718	PPP4R4	protein phosphatase 4, regulatory subunit 4	chr14:94640648-94746072	0.415569016	0.0187073	0.415283944	0.000638422
80755	AARSD1	alanyl-tRNA synthetase domain containing 1	chr17:41102441-41132545	0.416139748	0.00232257	0.442316204	0.00163713

7102	TSPAN7	tetraspanin 7	chrX:38420730-38548172	0.416353253	0.0452264	0.46888954	0.00116486
57628	DPP10	dipeptidyl-peptidase 10	chr2:115199898-116603807	0.416517784	0.00232257	0.405333129	0.000638422
22876	INPP5F	inositol polyphosphate-5-phosphatase F	chr10:121485558-121632394	0.417144752	0.00398798	0.318198734	0.000638422
3747	KCNC2	potassium voltage-gated channel, Shaw-related subfamily, member 2	chr12:75433895-75603581	0.417416635	0.00865834	0.329787815	0.000638422
26047	CNTNAP2	contactin associated protein-like 2	chr7:145813452-148118179	0.417633689	0.00654308	0.388942825	0.000638422
399947	C11orf87	chromosome 11 open reading frame 87	chr11:109292845-109299906	0.41817827	0.00232257	0.259693174	0.000638422
25797	QPCT	glutaminyl-peptide cyclotransferase	chr2:37552231-37600465	0.418407321	0.0199786	0.401310275	0.000638422
27145	FILIP1	filamin A interacting protein 1	chr6:75994726-76203496	0.418624891	0.0104723	0.441057911	0.00879958
9581	PREPL	prolyl endopeptidase-like	chr2:44502596-44589001	0.418772903	0.00962874	0.39542884	0.000638422
291	SLC25A4	solute carrier family 25 (mitochondrial carrier; adenine nucleotide translocator), member 4	chr4:186064416-186071538	0.419522473	0.00232257	0.498522319	0.000638422
80309	SPHKAP	SPHK1 interactor, AKAP domain containing	chr2:228844653-229046400	0.420795155	0.020736	0.263541122	0.000638422
7504	XK	X-linked Kx blood group (McLeod syndrome)	chrX:37545116-37591389	0.421782166	0.0221165	0.309407484	0.000638422
65055	REEP1	receptor accessory protein 1	chr2:86426444-86565206	0.423088096	0.00232257	0.345148711	0.0250929
3939	LDHA	lactate dehydrogenase A	chr11:18415935-18429765	0.42334918	0.0148125	0.400176957	0.000638422
7280	TUBB2A	tubulin, beta 2A	chr6:3153901-3157783	0.423915905	0.0104723	0.353560743	0.000638422
9331	B4GALT6	UDP-Gal:betaGlcNAc beta 1,4- galactosyltransferase, polypeptide 6	chr18:29201297-29268697	0.424736501	0.00232257	0.360209965	0.000638422
83698	CALN1	calneuron 1	chr7:71244469-71912292	0.425511492	0.00232257	0.38197232	0.000638422
84900	RNFT2	ring finger protein, transmembrane 2	chr12:117176095-117291436	0.426737264	0.00539633	0.411995362	0.000638422
84083	ZRANB3	zinc finger, RAN-binding domain containing 3	chr2:135954319-136288806	0.427113084	0.0382984	0.412158171	0.00625285
5864	RAB3A	RAB3A, member RAS oncogene family	chr19:18307610-18314874	0.427655204	0.0123074	0.225711214	0.000638422
1016	CDH18	cadherin 18, type 2	chr5:19466683-20288323	0.427806409	0.00654308	0.260836921	0.000638422
257194	NEGR1	neuronal growth regulator 1	chr1:71861249-72748277	0.428168332	0.00539633	0.304632126	0.000638422
30010	NXPH1	neurexophilin 1	chr7:8473584-8796074	0.428361285	0.0179767	0.384482543	0.000638422
84539	MCHR2	melanin-concentrating hormone receptor 2	chr6:100366083-100524295	0.428414733	0.00962874	0.379586345	0.000638422
93974	ATPIF1	ATPase inhibitory factor 1	chr1:28562601-28564616	0.429208336	0.01139	0.428325656	0.00563603
149111	CNIH3	cornichon homolog 3 (Drosophila)	chr1:224622312-224928337	0.429229161	0.016349	0.359770799	0.0386951
253152	EPHX4	epoxide hydrolase 4	chr1:92486955-92529202	0.429753114	0.0293492	0.382724987	0.00207151
4191	MDH2	malate dehydrogenase 2, NAD (mitochondrial)	chr7:75677364-75696828	0.430042157	0.0155725	0.418894835	0.000638422
8618	CADPS	Ca++-dependent secretion activator	chr3:62384019-62861064	0.430465643	0.0179767	0.37326962	0.000638422
6456	SH3GL2	SH3-domain GRB2-like 2	chr9:17578952-17797140	0.430895519	0.0131757	0.30654271	0.000638422
9804	TOMM20	translocase of outer mitochondrial membrane 20 homolog (yeast)	chr1:235272657-235292256	0.430928374	0.00539633	0.419638805	0.000638422
64168	NECAB1	N-terminal EF-hand calcium binding protein 1	chr8:91803920-91997485	0.431346753	0.0241004	0.420285037	0.000638422
1173	AP2M1	adaptor-related protein complex 2, mu 1 subunit	chr3:183892633-183901880	0.43160695	0.0221165	0.449735914	0.000638422
8867	SYNJ1	synaptojanin 1	chr21:34001068-34100351	0.432217682	0.00539633	0.438223878	0.000638422
163404	LPPR5	phosphatidic acid phosphatase type 2	chr1:99355800-99614408	0.432682297	0.0215169	0.43783828	0.00248927
57180	ACTR3B	ARP3 actin-related protein 3 homolog B (yeast)	chr7:152456850-152552737	0.433417707	0.00762148	0.457270578	0.000638422
81831	NETO2	neuropilin (NRP) and tolloid (TLL)-like 2	chr16:47109972-47177936	0.433597998	0.00762148	0.499639693	0.00396642
412	STS	steroid sulfatase (microsomal), isozyme S	chrX:6966960-7272683	0.433742284	0.00762148	0.392887997	0.00207151
219738	C10orf35	chromosome 10 open reading frame 35	chr10:71389976-71393361	0.434154367	0.0148125	0.376546516	0.000638422
10154	PLXNC1	plexin C1	chr12:94542498-94701451	0.434880221	0.0104723	0.481951541	0.000638422
379	ARL4D	ADP-ribosylation factor-like 4D	chr17:41476352-41478504	0.435399	0.0444129	0.366920649	0.00287725
309	ANXA6	annexin A6	chr5:150480266-150537795	0.435565019	0.00232257	0.322649568	0.000638422
490	ATP2B1	ATPase, Ca++ transporting, plasma membrane 1	chr12:89981825-90105729	0.436123912	0.0227801	0.44724895	0.000638422
122553	TRAPPC6B	trafficking protein particle complex 6B	chr14:39617014-39639634	0.436163213	0.00539633	0.482128627	0.000638422
2043	EPHA4	EPH receptor A4	chr2:222282654-222437010	0.436632069	0.00232257	0.417567114	0.000638422
260293	CYP4X1	cytochrome P450, family 4, subfamily X, polypeptide 1	chr1:47470012-47516426	0.43809632	0.0241004	0.380018089	0.00116486
10447	FAM3C	family with sequence similarity 3, member C	chr7:120988904-121036422	0.438254254	0.00232257	0.421902049	0.000638422
5213	PFKM	phosphofructokinase, muscle	chr12:48499655-48540187	0.438621976	0.00539633	0.499463099	0.000638422
55530	SVOP	SV2 related protein homolog (rat)	chr12:109302138-109458900	0.438880477	0.0139828	0.16728732	0.000638422
9607	CARTPT	CART prepropeptide	chr5:71014989-71016875	0.439480177	0.0199786	0.315228526	0.000638422
9867	PJA2	praja ring finger 2	chr5:108670339-108745708	0.439867221	0.01139	0.483236053	0.00361782
23229	ARHGEF9	Cdc42 guanine nucleotide exchange factor (GEF) 9	chrX:62854844-63005426	0.439909908	0.01139	0.362536963	0.000638422
226	ALDOA	aldolase A, fructose-bisphosphate	chr16:30064410-30081741	0.439934302	0.0227801	0.402918513	0.000638422
26033	ATRNL1	attractin-like 1	chr10:116852956-117708513	0.439976996	0.00398798	0.367954683	0.000638422
8507	ENC1	ectodermal-neural cortex (with BTB-like domain)	chr5:73923230-73937249	0.440504908	0.0392654	0.305190085	0.000638422
55055	ZWILCH	Zwilch, kinetochore associated, homolog (Drosophila)	chr15:66797430-66857835	0.440663711	0.037279	0.475131908	0.0328883
4008	LMO7	LIM domain 7	chr13:76194569-76434006	0.441403508	0.00398798	0.417110056	0.000638422
5126	PCSK2	proprotein convertase subtilisin/kexin type 2	chr20:17206751-17465225	0.441844307	0.00539633	0.329531892	0.000638422
4675	NAP1L3	nucleosome assembly protein 1-like 3	chrX:92925924-92928682	0.442558476	0.00398798	0.406985697	0.000638422
56934	CA10	carbonic anhydrase X	chr17:49707673-50301933	0.442671991	0.00539633	0.338923129	0.000638422
2567	GABRG3	gamma-aminobutyric acid (GABA) A receptor, gamma 3	chr15:27216174-27787131	0.442684265	0.0496454	0.302008278	0.000638422
374383	NCR3LG1	natural killer cell cytotoxicity receptor 3 ligand 1	chr11:17373308-17403430	0.442736432	0.0423787	0.363138047	0.000638422
3786	KCNQ3	potassium voltage-gated channel, KQT-like subfamily, member 3	chr8:133132585-133493004	0.443510449	0.00865834	0.385322944	0.000638422
84926	SPRYD3	SPRY domain containing 3	chr12:53436972-53473204	0.444153418	0.00962874	0.35539616	0.00879958
27075	TSPAN13	tetraspanin 13	chr7:16793350-16824250	0.445013189	0.0123074	0.488411813	0.000638422
202559	KHDRBS2	KH domain containing, RNA binding, signal transduction associated 2	chr6:62339665-62996100	0.445751019	0.0257447	0.368140914	0.00116486
26289	AK5	adenylate kinase 5	chr1:77747661-78149125	0.445924076	0.00654308	0.422586915	0.000638422
57181	SLC39A10	solute carrier family 39 (zinc transporter), member 10	chr2:196440618-196933536	0.44596426	0.020736	0.408701658	0.000638422
57084	SLC17A6	solute carrier family 17 (sodium-dependent inorganic phosphate cotransporter), member 6	chr11:22359666-22401046	0.446706762	0.0402926	0.441838182	0.00287725
22891	ZNF365	zinc finger protein 365	chr10:64099337-64431771	0.447497026	0.00762148	0.441122117	0.000638422
9515	STXBP5L	syntaxin binding protein 5-like	chr3:120627049-121143912	0.447773173	0.0246717	0.264481735	0.000638422
1641	DCX	doublecortin	chrX:110537006-110655460	0.447922176	0.00762148	0.437631958	0.00361782
145581	LRFN5	leucine rich repeat and fibronectin type III domain containing 5	chr14:42076763-42373752	0.448080547	0.0139828	0.307897061	0.000638422
2892	GRIA3	glutamate receptor, ionotrophic, AMPA 3	chrX:122318095-122624766	0.448099183	0.00232257	0.396048769	0.000638422
145773	FAM81A	family with sequence similarity 81, member A	chr15:59730371-59815751	0.448357054	0.0179767	0.297639932	0.000638422
2044	EPHA5	EPH receptor A5	chr4:66184867-66559104	0.448686599	0.0192412	0.262887965	0.000638422
64881	PCDH20	protocadherin 20	chr13:61982674-61989655	0.448839018	0.00962874	0.392036531	0.000638422
8775	NAPA	N-ethylmaleimide-sensitive factor attachment protein, alpha	chr19:47987538-48018515	0.44914401	0.00398798	0.469461905	0.000638422
51400	PPME1	protein phosphatase methylesterase 1	chr11:73882337-73965754	0.449648637	0.00398798	0.387530026	0.000638422
57172	CAMK1G	calcium/calmodulin-dependent protein kinase IG	chr1:209757044-209787284	0.449985369	0.00762148	0.257477809	0.000638422
79698	ZMAT4	zinc finger, matrin type 4	chr8:40377098-40755343	0.451525697	0.0286978	0.272780042	0.000638422
57451	ODZ2	odz, odd Oz/ten-m homolog 2 (Drosophila)	chr5:166711842-167696407	0.452537734	0.0148125	0.38365727	0.000638422
2903	GRIN2A	glutamate receptor, ionotropic, N-methyl D-aspartate 2A	chr16:9660366-10277366	0.453822486	0.0155725	0.310860669	0.000638422
57613	KIAA1467	KIAA1467	chr12:13175053-13256630	0.453866528	0.00539633	0.384256082	0.000638422
891	CCNB1	cyclin B1	chr5:68462912-68474070	0.454168641	0.0277378	0.488750472	0.0203437
506	ATP5B	ATP synthase, H+ transporting, mitochondrial F1 complex, beta polypeptide	chr12:57031958-57039852	0.454370161	0.0263821	0.472628981	0.000638422
84867	PTPN5	protein tyrosine phosphatase, non-receptor type 5 (striatum-enriched)	chr11:18749472-18813389	0.454663155	0.0330887	0.315983252	0.000638422
51209	RAB9B	RAB9B, member RAS oncogene family	chrX:103077254-103087212	0.454820756	0.00865834	0.422481478	0.000638422
340543	TCEAL5	transcription elongation factor A (SII)-like 5	chrX:102528610-102531797	0.454890118	0.01139	0.380439777	0.000638422
27012	KCNV1	potassium channel, subfamily V, member 1	chr8:110975498-110988062	0.455050953	0.0171483	0.237289472	0.000638422
114134	SLC2A13	solute carrier family 2 (facilitated glucose transporter), member 13	chr12:40148822-40499853	0.45527811	0.00962874	0.372605274	0.000638422
5530	PPP3CA	protein phosphatase 3 (formerly 2B), catalytic subunit, alpha isoform	chr4:101944572-102270665	0.455830701	0.0227801	0.433570949	0.000638422
64919	BCL11B	B-cell CLL/lymphoma 11B (zinc finger protein)	chr14:99635624-99737822	0.455969743	0.0139828	0.473081287	0.00432944
57446	NDRG3	NDRG family member 3	chr20:35280167-35374541	0.456219494	0.00232257	0.494728871	0.000638422
222008	VSTM2A	V-set and transmembrane domain containing 2A	chr7:54609395-54642450	0.456393453	0.00654308	0.403594942	0.000638422
10873	ME3	malic enzyme 3, NADP(+)-dependent, mitochondrial	chr11:86145968-86383678	0.456605455	0.0293492	0.310197723	0.000638422
27067	STAU2	staufen, RNA binding protein, homolog 2 (Drosophila)	chr8:74332308-74659943	0.456757398	0.0104723	0.38024996	0.000638422
2744	GLS	glutaminase	chr2:191745537-191879003	0.457593987	0.0192412	0.471725665	0.0101566
5188	PET112	PET112 homolog (yeast)	chr4:152591808-152682175	0.457609846	0.00865834	0.489336906	0.00248927
340533	KIAA2022	KIAA2022	chrX:73952687-74145287	0.457777988	0.0148125	0.41612821	0.000638422
10752	CHL1	cell adhesion molecule with homology to L1CAM (close homolog of L1)	chr3:238649-451224	0.458174795	0.0104723	0.483416962	0.000638422

5218	CDK14	PFTAIRE protein kinase 1	chr7:90225910-90839963	0.458200202	0.00762148	0.333008291	0.000638422
5865	RAB3B	RAB3B, member RAS oncogene family	chr1:52373627-52456436	0.459532881	0.0139828	0.216916244	0.000638422
387856	C12orf68	chromosome 12 open reading frame 68	chr12:48577365-48579709	0.459558364	0.0378873	0.253323004	0.000638422
338811	FAM19A2	family with sequence similarity 19 (chemokine (C-C motif)-like), member A2	chr12:62080249-62586551	0.459994973	0.0263821	0.411544403	0.000638422
1949	EFNB3	ephrin-B3	chr17:7608228-7614708	0.460294784	0.00539633	0.307897061	0.000638422
55312	RFK	riboflavin kinase	chr9:79000432-79009445	0.460837492	0.00654308	0.42637655	0.000638422
84303	CHCHD6	coiled-coil-helix-coiled-coil-helix domain containing 6	chr3:126423070-126679317	0.460955696	0.0315465	0.36437604	0.000638422
79442	LRRC2	leucine rich repeat containing 2	chr3:46556664-46608011	0.46219386	0.0123074	0.463200912	0.00361782
8936	WASF1	WAS protein family, member 1	chr6:110421021-110501237	0.464203723	0.00762148	0.390377011	0.000638422
2562	GABRB3	gamma-aminobutyric acid (GABA) A receptor, beta 3	chr15:26788693-27018935	0.46521193	0.00762148	0.352339958	0.000638422
81706	PPP1R14C	protein phosphatase 1, regulatory (inhibitor) subunit 14C	chr6:150464187-150571528	0.465705556	0.0282007	0.334405389	0.000638422
10953	TOMM34	translocase of outer mitochondrial membrane 34	chr20:43538702-43589114	0.466238485	0.00654308	0.467588061	0.00465363
844	CASQ1	calsequestrin 1 (fast-twitch, skeletal muscle)	chr1:160160284-160171676	0.466413031	0.0263821	0.399539487	0.000638422
8237	USP11	ubiquitin specific peptidase 11	chrX:47092313-47107730	0.466784966	0.0123074	0.372574283	0.000638422
25758	C11orf41	chromosome 11 open reading frame 41	chr11:33398175-33695648	0.466788201	0.0104723	0.378068616	0.000638422
548596	CKMT1A	creatine kinase, mitochondrial 1A	chr15:43985083-43991420	0.467157197	0.0465332	0.316055538	0.000638422
3382	ICA1	islet cell autoantigen 1, 69kDa	chr7:8152814-8302450	0.467215486	0.0221165	0.278044942	0.000638422
3763	KCNJ6	potassium inwardly-rectifying channel, subfamily J, member 6	chr21:38979529-39290751	0.468395785	0.0277378	0.480633796	0.000638422
23710	GABARAPL1	GABA(A) receptor-associated protein like 1	chr12:10365488-10375724	0.468746557	0.0257447	0.373515495	0.000638422
4747	NEFL	neurofilament, light polypeptide	chr8:24808466-24814131	0.469461905	0.0330887	0.283330928	0.000638422
261729	STEAP2	six transmembrane epithelial antigen of the prostate 2	chr7:89840999-89867310	0.470106652	0.016349	0.471532789	0.00116486
23236	PLCB1	phospholipase C, beta 1 (phosphoinositide-specific)	chr20:8113295-8865547	0.471127679	0.00962874	0.342391787	0.000638422
5602	MAPK10	mitogen-activated protein kinase 10	chr4:86396283-87374504	0.471255055	0.0496454	0.483055212	0.0120438
54769	DIRAS2	DIRAS family, GTP-binding RAS-like 2	chr9:93372113-93405108	0.47135306	0.0227801	0.327255649	0.000638422
777	CACNA1E	calcium channel, voltage-dependent, R type, alpha 1E subunit	chr1:181452019-181777624	0.471748554	0.00232257	0.490433693	0.000638422
55074	OXR1	oxidation resistance 1	chr8:107282405-107764921	0.47206257	0.0192412	0.430447741	0.000638422
50813	COPS7A	COP9 constitutive photomorphogenic homolog subunit 7A (Arabidopsis)	chr12:6833149-6841041	0.472088747	0.00962874	0.438609815	0.000638422
54602	NDFIP2	Nedd4 family interacting protein 2	chr13:80055258-80130214	0.472098564	0.00654308	0.455596952	0.000638422
9495	AKAP5	A kinase (PRKA) anchor protein 5	chr14:64932216-64941221	0.473035381	0.00962874	0.341962493	0.000638422
133308	SLC9B2	solute carrier family 9, subfamily B (NHA2, cation proton antiporter 2), member 2	chr4:103942720-103998502	0.473055054	0.0221165	0.416148401	0.000638422
11040	PIM2	pim-2 oncogene	chrX:48770458-48776413	0.47306817	0.0263821	0.428866342	0.00116486
84302	C9orf125	chromosome 9 open reading frame 125	chr9:104228642-104266667	0.473189511	0.00654308	0.419592269	0.000638422
285175	UNC80	chromosome 2 open reading frame 21	chr2:210636716-210864137	0.473557003	0.0286978	0.435046043	0.00116486
10313	RTN3	reticulon 3	chr11:63448921-63527363	0.473642354	0.0492625	0.452158346	0.00684201
1741	DLG3	discs, large homolog 3 (Drosophila)	chrX:69664704-69725340	0.474128493	0.0123074	0.371383082	0.000638422
9500	MAGED1	melanoma antigen family D, 1	chrX:51546154-51645454	0.474253393	0.0123074	0.410635426	0.000638422
5601	MAPK9	mitogen-activated protein kinase 9	chr5:179660594-179719071	0.474467114	0.00962874	0.445550233	0.000638422
219749	ZNF25	zinc finger protein 25	chr10:38238794-38265453	0.474934347	0.00762148	0.452506368	0.000638422
808	CALM3	calmodulin 3 (phosphorylase kinase, delta)	chr19:47104511-47114053	0.476318999	0.0192412	0.319281308	0.000638422
9348	NDST3	N-deacetylase/N-sulfotransferase (heparan glucosaminyl) 3	chr4:118954438-119179789	0.476735181	0.0192412	0.304461139	0.000638422
8508	NIPSNAP1	nipsnap homolog 1 (C. elegans)	chr22:29950797-29977326	0.477555396	0.01139	0.419042942	0.000638422
4591	TRIM37	tripartite motif-containing 37	chr17:56833231-57184266	0.477661333	0.0387574	0.363651895	0.000638422
84553	C6orf168	chromosome 6 open reading frame 168	chr6:99718654-99797857	0.478426763	0.0192412	0.362320917	0.000638422
3751	KCND2	potassium voltage-gated channel, Shal-related subfamily, member 2	chr7:119913721-120390387	0.479136957	0.01139	0.471611237	0.00116486
51155	HN1	hematological and neurological expressed 1	chr17:73131343-73150775	0.479249888	0.016349	0.378994811	0.000638422
79750	ZNF385D	zinc finger protein 385D	chr3:21457766-21792816	0.480600482	0.0253156	0.396507483	0.0211951
5646	PRSS3	protease, serine, 3	chr9:33750463-33799229	0.481464055	0.00962874	0.443559639	0.00248927
203859	ANO5	anoctamin 5	chr11:22214721-22305647	0.481924817	0.0139828	0.457914451	0.000638422
5581	PRKCE	protein kinase C, epsilon	chr2:45879042-46415129	0.482452897	0.0171483	0.326489839	0.000638422
348980	HCN1	hyperpolarization activated cyclic nucleotide-gated potassium channel 1	chr5:45255043-45696570	0.482660276	0.0270075	0.289292334	0.000638422
144165	PRICKLE1	prickle homolog 1 (Drosophila)	chr12:42850557-42983572	0.482780731	0.0215169	0.385071966	0.000638422
158931	TCEAL6	transcription elongation factor A (SII)-like 6	chrX:101394932-101399134	0.483323149	0.0257447	0.357038115	0.000638422
55190	NUDT11	nudix (nucleoside diphosphate linked moiety X)-type motif 11	chrX:51232862-51241746	0.483376754	0.0341672	0.337334084	0.000638422
64084	CLSTN2	calsyntenin 2	chr3:139653971-140296237	0.484003706	0.0402926	0.379681076	0.000638422
440270	GOLGA8B	golgin A8 family, member B	chr15:34671244-34875879	0.484779299	0.0347863	0.457020251	0.0239279
10645	CAMKK2	calcium/calmodulin-dependent protein kinase kinase 2, beta	chr12:121675315-121736111	0.487451302	0.0148125	0.324686656	0.000638422
783	CACNB2	calcium channel, voltage-dependent, beta 2 subunit	chr10:18429605-18832429	0.488235803	0.0419389	0.469803706	0.000638422
50650	ARHGEF3	Rho guanine nucleotide exchange factor (GEF) 3	chr3:56761445-57113336	0.48835765	0.0253156	0.380466148	0.000638422
25927	CNRIP1	cannabinoid receptor interacting protein 1	chr2:68511302-68547183	0.488465983	0.0155725	0.489594752	0.000638422
3756	KCNH1	potassium voltage-gated channel, subfamily H (eag-related), member 1	chr1:210501595-211307470	0.48856418	0.0378873	0.319957014	0.000638422
51046	ST8SIA3	ST8 alpha-N-acetyl-neuraminide alpha-2,8-sialyltransferase 3	chr18:55019643-55036448	0.489903669	0.0305778	0.331426301	0.000638422
2561	GABRB2	gamma-aminobutyric acid (GABA) A receptor, beta 2	chr5:160715419-160975130	0.490556087	0.0315465	0.300763208	0.000638422
4978	OPCML	opioid binding protein/cell adhesion molecule-like	chr11:132284680-133402403	0.492047671	0.0419389	0.33664268	0.000638422
1141	CHRNB2	cholinergic receptor, nicotinic, beta 2 (neuronal)	chr1:154540254-154553650	0.494194205	0.0456847	0.26574418	0.000638422
3831	KLC1	kinesin light chain 1	chr14:104095477-104181823	0.494506024	0.0310303	0.461284909	0.000638422
56950	SMYD2	SET and MYND domain containing 2	chr1:214454472-214510492	0.495586914	0.0392654	0.414697139	0.000638422
9456	HOMER1	homer homolog 1 (Drosophila)	chr5:78668614-78810309	0.495758702	0.0199786	0.393831387	0.000638422
151126	ZNF385B	zinc finger protein 385B	chr2:180306708-180726232	0.495923673	0.0328287	0.347404898	0.000638422
164832	LONRF2	LON peptidase N-terminal domain and ring finger 2	chr2:100888337-100939633	0.496463651	0.0131757	0.460454338	0.000638422
56675	NRIP3	nuclear receptor interacting protein 3	chr11:9001966-9026073	0.497276445	0.0192412	0.269265257	0.000638422
23508	TTC9	tetratricopeptide repeat domain 9	chr14:71108503-71142077	0.497493644	0.0458453	0.419054561	0.000638422
2122	MECOM	ecotropic viral integration site 1	chr3:168801282-169381563	2.012334309	0.043185	3.171114566	0.000638422
168537	GIMAP7	GTPase, IMAP family member 7	chr7:150211944-150218161	2.015293552	0.0246717	2.151540504	0.0091
11177	BAZ1A	bromodomain adjacent to zinc finger domain, 1A	chr14:35221936-35345725	2.040637608	0.0241004	2.91575601	0.000638422
64344	HIF3A	hypoxia inducible factor 3, alpha subunit	chr19:46800302-46846691	2.041090286	0.0362874	3.47913823	0.000638422
100463482	MTRNR2L6	MT-RNR2-like 6	chr7:142364653-142381919	2.055928369	0.0423787	2.014734871	0.00465363
5613	PRKX	protein kinase, X-linked	chrX:3522383-3631675	2.125098923	0.0263821	4.10163752	0.000638422
4256	MGP	matrix Gla protein	chr12:14956463-15091463	2.131309337	0.0263821	4.523250828	0.000638422
27106	ARRDC2	arrestin domain containing 2	chr19:18111943-18124911	2.161825387	0.0367197	2.720081729	0.000638422
8416	ANXA9	annexin A9	chr1:150954498-150968114	2.169511149	0.0282007	2.528286577	0.00825241
55114	ARHGAP17	Rho GTPase activating protein 17	chr16:24930614-25189559	2.184813298	0.0310303	2.372536036	0.0321922
64123	ELTD1	EGF, latrophilin and seven transmembrane domain containing 1	chr1:79355448-79472495	2.190332663	0.0465332	2.462732616	0.000638422
79660	PPP1R3B	protein phosphatase 1, regulatory (inhibitor) subunit 3B	chr8:8993763-9009152	2.224390375	0.0187073	2.277782509	0.00361782
116441	TM4SF18	transmembrane 4 L six family member 18	chr3:149036284-149051548	2.240079602	0.00539633	2.624131468	0.000638422
100505495	PCAT19	prostate cancer associated transcript 19 (non-protein coding)	chr19:41960073-42007111	2.271050836	0.037279	2.341819692	0.00799412
5364	PLXNB1	plexin B1	chr3:48445260-48471460	2.334510375	0.00654308	2.497128391	0.000638422
342892	ZNF850	zinc finger protein 850	chr19:37234286-37263721	2.361494213	0.00865834	2.26677312	0.0278078
3075	CFH	complement factor H	chr1:196620940-196716636	2.39813022	0.0131757	2.620023951	0.000638422
9510	ADAMTS1	ADAM metallopeptidase with thrombospondin type 1 motif, 1	chr21:28208557-28217756	2.415865271	0.00654308	3.079892508	0.000638422
4069	LYZ	lysozyme (renal amyloidosis)	chr12:69742133-69748013	2.428743273	0.00654308	2.031196762	0.0257176
9976	CLEC2B	C-type lectin domain family 2, member B	chr12:10004967-10022458	2.433596528	0.00654308	2.245645188	0.0170267
10749	KIF1C	kinesin family member 1C	chr17:4900952-4931694	2.495138675	0.00232257	5.082858258	0.000638422
54751	FBLIM1	filamin binding LIM protein 1	chr1:16084824-16113084	2.533496788	0.00654308	2.354547782	0.0182601
7043	TGFB3	transforming growth factor, beta 3	chr14:76424441-76448594	2.538401009	0.00654308	4.261761919	0.000638422
4487	MSX1	msh homeobox 1	chr4:4861391-4865660	2.542257209	0.00398798	3.311970907	0.000638422
1278	COL1A2	collagen, type I, alpha 2	chr7:94023872-94060544	2.579711979	0.0270075	3.789532816	0.000638422
51365	PLA1A	phospholipase A1 member A	chr3:119316694-119348660	2.610941259	0.0253156	3.057261591	0.000638422
7130	TNFAIP6	tumor necrosis factor, alpha-induced protein 6	chr2:152214105-152236562	2.620260049	0.00539633	2.568667227	0.0101566
72	ACTG2	actin, gamma 2, smooth muscle, enteric	chr2:74120092-74146780	2.621786123	0.0234694	4.335174301	0.000638422
2669	GEM	GTP binding protein overexpressed in skeletal muscle	chr8:95261484-95274547	2.6899512	0.01139	2.28963876	0.00432944
4500	MT1L	metallothionein 1L (gene/pseudogene)	chr16:56650899-56652737	2.746567979	0.00398798	3.714633819	0.00116486
7169	TPM2	tropomyosin 2 (beta)	chr9:35681989-35691108	2.776043164	0.0187073	2.114094695	0.00163713

**25959**	**KANK2**	**KN motif and ankyrin repeat domains 2**	**chr19:11274791-11308243**	**2.812708414**	**0.00232257**	**3.056202208**	**0.000638422**
**58475**	**MS4A7**	**membrane-spanning 4-domains, subfamily A, member 7**	**chr11:60145957-60185228**	**2.909860517**	**0.00654308**	**2.374609032**	**0.00163713**
**6876**	**TAGLN**	**transgelin**	**chr11:117070039-117075508**	**2.918748695**	**0.00232257**	**2.465585017**	**0.000638422**
**9457**	**FHL5**	**four and a half LIM domains 5**	**chr6:97010423-97071054**	**2.923729822**	**0.00654308**	**10.50620693**	**0.000638422**
**2828**	**GPR4**	**G protein-coupled receptor 4**	**chr19:46093022-46105466**	**3.075902983**	**0.00962874**	**2.233289068**	**0.00287725**
**150946**	**FAM59B**	**family with sequence similarity 59, member B**	**chr2:26395959-26413241**	**3.093628585**	**0.00232257**	**2.901783731**	**0.000638422**
**2872**	**MKNK2**	**MAP kinase interacting serine/threonine kinase 2**	**chr19:2037463-2051243**	**3.139881255**	**0.00762148**	**2.229793331**	**0.00287725**
**283755**	**HERC2P3**	**hect domain and RLD 2 pseudogene 3**	**chr15:20586683-20711433**	**3.172697556**	**0.00232257**	**6.604594557**	**0.000638422**
**134549**	**SHROOM1**	**shroom family member 1**	**chr5:132157663-132166703**	**3.256833576**	**0.00232257**	**2.504877412**	**0.000638422**
**353514**	**LILRA5**	**leukocyte immunoglobulin-like receptor, subfamily A (with TM domain), member 5**	**chr19:54818352-54824409**	**3.294272731**	**0.00232257**	**6.066986075**	**0.00396642**
**59**	**ACTA2**	**actin, alpha 2, smooth muscle, aorta**	**chr10:90694830-90777253**	**3.402251992**	**0.00865834**	**2.914806272**	**0.00825241**
**845**	**CASQ2**	**calsequestrin 2 (cardiac muscle)**	**chr1:116242519-116345204**	**3.491410523**	**0.00962874**	**5.322706439**	**0.000638422**
**23704**	**KCNE4**	**potassium voltage-gated channel, Isk-related family, member 4**	**chr2:223916861-223920355**	**4.436801472**	**0.00232257**	**4.699574041**	**0.000638422**
**6279**	**S100A8**	**S100 calcium binding protein A8**	**chr1:153362507-153363664**	**5.018965399**	**0.00232257**	**2.142150734**	**0.0151369**
**871**	**SERPINH1**	**serpin peptidase inhibitor, clade H (heat shock protein 47), member 1, (collagen binding protein 1)**	**chr11:75273100-75283870**	**5.437863735**	**0.00232257**	**2.129345418**	**0.0101566**
**6275**	**S100A4**	**S100 calcium binding protein A4**	**chr1:153516094-153518282**	**5.52659158**	**0.00232257**	**4.606560625**	**0.000638422**
**26266**	**SLC13A4**	**solute carrier family 13 (sodium/sulfate symporters), member 4**	**chr7:135365246-135412933**	**6.580968881**	**0.00232257**	**2.996233377**	**0.000638422**
**3303**	**HSPA1A**	**heat shock 70kDa protein 1A**	**chr6:31777395-31785719**	**6.599057552**	**0.00232257**	**3.8880048**	**0.000638422**
**3304**	**HSPA1B**	**heat shock 70kDa protein 1B**	**chr6:31795511-31798031**	**6.599057552**	**0.00232257**	**2.366327899**	**0.000638422**
**375061**	**FAM89A**	**family with sequence similarity 89, member A**	**chr1:231154703-231175995**	**8.186858725**	**0.00232257**	**2.253222839**	**0.00248927**
**100500849**	**MIR3916**	**microRNA 3916**	**chr1:247342111-247374105**	**17.2291545**	**0.00762148**	**2.127294836**	**0.00287725**

The core set of 522 DEGs in the frontal cortex of AD overlapping between EMU and UKY satisfying q-Value (FDR-corrected p-Value) < 0.05 and fold change greater than 2.0 or smaller than 0.5 were extracted by RNA-Seq data analysis of SRA060572. They are listed with Entrez Gene ID, Gene Symbol, Gene Name, Chromosomal Locus, Fold Change, and q-Value. NEUROD6 is underlined.

Supplementary TABLE 2. The set of 60 differentially spliced genes in the frontal cortex of AD identified by RNA-Seq data analysis of SRA060752

Entrez Gene ID	Gene Symbol	Gene Name	Chromosome Locus	Sqrt JS	q-Value
5797	PTPRM	protein tyrosine phosphatase, receptor type, M	chr18:7567313-8406952	0.825781	0.0142952
5468	PPARG	peroxisome proliferator-activated receptor gamma	chr3:12329348-12476009	0.735183	0.0142952
2635	GBP3	guanylate binding protein 3	chr1:89472359-89488549	0.727772	0.035452
7516	XRCC2	X-ray repair complementing defective repair in Chinese hamster cells 2	chr7:152341056-152373250	0.721052	0.0142952
23348	DOCK9	dedicator of cytokinesis 9	chr13:99445739-99738687	0.712575	0.0142952
441869	LOC441869	hypothetical protein LOC441869	chr1:1352433-1357201	0.712559	0.0142952
94121	SYTL4	synaptotagmin-like 4	chrX:99929488-99987135	0.706969	0.0208722
5163	PDK1	pyruvate dehydrogenase kinase, isozyme 1	chr2:173420640-173464990	0.68768	0.0142952
23332	CLASP1	cytoplasmic linker associated protein 1	chr2:122095164-122407091	0.68699	0.035452
25788	RAD54B	RAD54 homolog B (S. cerevisiae)	chr8:95383956-95487343	0.676423	0.0302148
26091	HERC4	hect domain and RLD 4	chr10:69681655-69835103	0.638866	0.0142952
144406	WDR66	WD repeat domain 66	chr12:122356462-122441832	0.626485	0.0142952
26034	IPCEF1	interaction protein for cytohesin exchange factors 1	chr6:154331635-154677900	0.624585	0.0142952
27124	INPP5J	inositol polyphosphate-5-phosphatase J	chr22:31518727-31530683	0.61534	0.0142952
51454	GULP1	GULP, engulfment adaptor PTB domain containing 1	chr2:189156388-189460669	0.605783	0.0142952
57125	PLXDC1	plexin domain containing 1	chr17:37213271-37309211	0.586646	0.0142952
57476	GRAMD1B	GRAM domain containing 1B	chr11:123396297-123498480	0.58641	0.0142952
221	ALDH3B1	aldehyde dehydrogenase 3 family, member B1	chr11:67776047-67796852	0.580375	0.035452
55036	CCDC40	coiled-coil domain containing 40	chr17:78010430-78074468	0.576498	0.0142952
8120	AP3B2	adaptor-related protein complex 3, beta 2 subunit	chr15:83211862-83378635	0.554378	0.0142952
11176	BAZ2A	bromodomain adjacent to zinc finger domain, 2A	chr12:56915608-57030163	0.552978	0.0208722
158471	PRUNE2	prune homolog 2 (Drosophila)	chr9:79224615-79521133	0.549799	0.0142952
1503	CTPS	CTP synthase	chr1:41445006-41478403	0.546957	0.0142952
353189	SLCO4C1	solute carrier organic anion transporter family, member 4C1	chr5:101569536-101632253	0.544079	0.0142952
6599	SMARCC1	SWI/SNF related, matrix associated, actin dependent regulator of chromatin, subfamily c, member 1	chr3:47627377-47823405	0.524068	0.0208722
79000	AUNIP	aurora kinase A and ninein interacting protein	chr1:26159495-26185869	0.520829	0.0142952
606495	CYB5RL	cytochrome b5 reductase-like	chr1:54635094-54665746	0.504141	0.035452
6596	HLTF	helicase-like transcription factor	chr3:148747775-148804341	0.488028	0.0208722
23114	NFASC	neurofascin homolog (chicken)	chr1:204797781-204991955	0.476222	0.0302148
10919	EHMT2	euchromatic histone-lysine N-methyltransferase 2	chr6:31847534-31865464	0.468069	0.0142952
6829	SUPT5H	suppressor of Ty 5 homolog (S. cerevisiae)	chr19:39936185-39967308	0.467546	0.0142952
1454	CSNK1E	casein kinase 1, epsilon	chr22:38686650-38714174	0.464769	0.0216171
26038	CHD5	chromodomain helicase DNA binding protein 5	chr1:6161840-6240218	0.4647	0.0216171
165918	RNF168	ring finger protein 168	chr3:196195656-196230639	0.455392	0.0208722
64324	NSD1	nuclear receptor binding SET domain protein 1	chr5:176559969-176727214	0.448436	0.0142952
9256	BZRAP1	benzodiazapine receptor (peripheral) associated protein 1	chr17:56378585-56494931	0.422966	0.035452
57478	USP31	ubiquitin specific peptidase 31	chr16:23072727-23160591	0.416776	0.0142952
80255	SLC35F5	solute carrier family 35, member F5	chr2:114464233-114514862	0.416096	0.0142952
9760	TOX	thymocyte selection-associated high mobility group box	chr8:59717971-60031855	0.415069	0.0142952
57631	LRCH2	leucine-rich repeats and calponin homology (CH) domain containing 2	chrX:114345175-114468635	0.411526	0.0302148
55183	RIF1	RAP1 interacting factor homolog (yeast)	chr2:152266396-152334001	0.407443	0.0208722
284161	GDPD1	glycerophosphodiester phosphodiesterase domain containing 1	chr17:57297827-57353330	0.40268	0.0208722
26052	DNM3	dynamin 3	chr1:171810620-172381857	0.397641	0.0216171
440823	MIAT	myocardial infarction associated transcript (non-protein coding)	chr22:27042837-27072441	0.389809	0.0208722
6645	SNTB2	syntrophin, beta 2 (dystrophin-associated protein A1, 59kDa, basic component 2)	chr16:69221049-69342955	0.389015	0.0216171
84864	MINA	MYC induced nuclear antigen	chr3:97540880-97691295	0.385104	0.035452
284677	C1orf204	chromosome 1 open reading frame 204	chr1:159796478-159832447	0.371537	0.0208722
391123	VSIG8	V-set and immunoglobulin domain containing 8	chr1:159796478-159832447	0.371537	0.0208722
6489	ST8SIA1	ST8 alpha-N-acetyl-neuraminide alpha-2,8-sialyltransferase 1	chr12:22346281-22487648	0.370318	0.0142952
29801	ZDHHC8	zinc finger, DHHC-type containing 8	chr22:20119363-20135632	0.364549	0.0216171
9321	TRIP11	thyroid hormone receptor interactor 11	chr14:92432265-92511291	0.364451	0.0142952
9631	NUP155	nucleoporin 155kDa	chr5:37286396-37371197	0.351752	0.0142952
57120	GOPC	golgi associated PDZ and coiled-coil motif containing	chr6:117803819-117923705	0.347199	0.0142952
55362	TMEM63B	transmembrane protein 63B	chr6:44095333-44123256	0.332719	0.0216171
23028	KDM1A	lysine (K)-specific demethylase 1	chr1:23345940-23410184	0.327325	0.0216171
23600	AMACR	alpha-methylacyl-CoA racemase	chr5:33987090-34124633	0.307388	0.0142952
5358	PLS3	plastin 3 (T isoform)	chrX:114795176-114885179	0.303812	0.0216171
55763	EXOC1	exocyst complex component 1	chr4:56719815-56771244	0.303033	0.0216171
80218	NAA50	N-acetyltransferase 13 (GCN5-related)	chr3:113432171-113465120	0.248626	0.0142952
23276	KLHL18	kelch-like 18 (Drosophila)	chr3:47205859-47392593	0.242977	0.0216171

Differentially spliced genes in the frontal cortex compared between AD versus NC derived from EMU and UKY extracted by RNA-Seq data analysis of SRA060572 were combined. The spliced genes satisfying q-Value (FDR-corrected p-Value) < 0.05 are listed with Entrez Gene ID, Gene Symbol, Gene Name, Chromosomal Locus, the square root of the Jensen–Shannon divergence (Sqrt JS), and q-Value.

Supplementary TABLE 3. The set of 215 DEGs in cortical neurons of the superior frontal gyrus of AD identified by microarray data analysis of GSE5281

Entrez Gene ID	Gene Symbol	Gene Title	Fold Change (AD vs NC)	p-Value
116	ADCYAP1	adenylate cyclase activating polypeptide 1 (pituitary)	0.069636923	1.26039E-07
6750	SST	somatostatin	0.078547047	2.49899E-05
25859	PART1	prostate androgen-regulated transcript 1 (non-protein coding)	0.09979003	7.81005E-07
10556	RPP30	ribonuclease P/MRP 30kDa subunit	0.121421574	1.15361E-05
7306	TYRP1	tyrosinase-related protein 1	0.12391224	2.91523E-06
729870	LOC729870	uncharacterized LOC729870	0.125916106	1.14639E-07
10001	MED6	mediator complex subunit 6	0.127676215	1.04388E-05
5771	PTPN2	protein tyrosine phosphatase, non-receptor type 2	0.129512873	1.61631E-05
126068	ZNF441	zinc finger protein 441	0.135568912	3.65191E-06
9040	UBE2M	ubiquitin-conjugating enzyme E2M (UBC12 homolog, yeast)	0.140010654	9.52306E-06
2563	GABRD	gamma-aminobutyric acid (GABA) A receptor, delta	0.142042327	2.57797E-05
57452	GALNT16	polypeptide N-acetylgalactosaminyltransferase 16	0.143744955	8.22479E-06
1412	CRYBA2	crystallin, beta A2	0.145842233	9.60478E-05
53918	PELO	pelota homolog (Drosophila)	0.147274585	3.97322E-05
10777	ARPP21	cAMP-regulated phosphoprotein, 21kDa	0.1474134	4.52823E-06
64105	CENPK	centromere protein K	0.148647796	2.45922E-05
158228	FAM201A	family with sequence similarity 201, member A	0.149625358	3.40468E-08
196294	IMMP1L	IMP1 inner mitochondrial membrane peptidase-like (S. cerevisiae)	0.15002806	1.03281E-05
158326	FREM1	FRAS1 related extracellular matrix 1	0.15200796	2.39567E-07
283401	FLJ33996	Hypothetical protein FLJ33996	0.154423179	2.77206E-05
8754	ADAM9	ADAM metallopeptidase domain 9	0.154955285	3.00411E-07
6511	SLC1A6	solute carrier family 1 (high affinity aspartate/glutamate transporter), member 6	0.156759363	4.74541E-05
5605	MAP2K2	mitogen-activated protein kinase kinase 2	0.157368364	6.95049E-06
51230	PHF20	PHD finger protein 20	0.157471386	2.87845E-05
5600	MAPK11	mitogen-activated protein kinase 11	0.16197929	3.94138E-08
81573	ANKRD13C	ankyrin repeat domain 13C	0.165393484	9.22967E-05
221264	AKD1	adenylate kinase domain containing 1	0.167076883	2.44999E-05
8539	API5	apoptosis inhibitor 5	0.169466	6.32302E-05
57379	AICDA	activation-induced cytidine deaminase	0.171641877	6.25246E-07
728192	LINC00460	long intergenic non-protein coding RNA 460	0.171656016	8.49002E-07
201626	PDE12	Phosphodiesterase 12	0.172443023	1.6E-05
23304	UBR2	ubiquitin protein ligase E3 component n-recognin 2	0.17290985	4.0324E-05
90632	C6orf176	chromosome 6 open reading frame 176	0.174179808	1.05826E-05
256471	MFSD8	major facilitator superfamily domain containing 8	0.178004149	3.08466E-06
1846	DUSP4	dual specificity phosphatase 4	0.180708065	3.17436E-05
56915	EXOSC5	exosome component 5	0.18089212	4.4006E-05
81602	CDADC1	cytidine and dCMP deaminase domain containing 1	0.181221497	5.63843E-06
22843	PPM1E	protein phosphatase, Mg2+/Mn2+ dependent, 1E	0.181354038	9.88435E-05
891	CCNB1	cyclin B1	0.182817517	1.34014E-05
2762	GMDS	GDP-mannose 4,6-dehydratase	0.183086528	3.97601E-06
7062	TCHH	trichohyalin	0.183269147	9.68165E-06
6835	SURF2	surfeit 2	0.184390125	3.26572E-05
81491	GPR63	G protein-coupled receptor 63	0.186613153	3.74939E-05
55172	C14orf104	chromosome 14 open reading frame 104	0.186734612	8.42116E-07
3914	LAMB3	laminin, beta 3	0.189677043	4.52811E-06
523	ATP6V1A	ATPase, H+ transporting, lysosomal 70kDa, V1 subunit A	0.190442377	8.45909E-05
51086	TNNI3K	TNNI3 interacting kinase	0.192973193	1.79598E-05
169200	TMEM64	transmembrane protein 64	0.193218848	4.05358E-06
9734	HDAC9	histone deacetylase 9	0.19323932	1.28179E-05
79693	YRDC	yrdC domain containing (E. coli)	0.193557047	8.18589E-07
4801	NFYB	nuclear transcription factor Y, beta	0.194719115	3.02116E-05
93426	SYCE1	synaptonemal complex central element protein 1	0.195656453	1.51885E-05
84520	C14orf142	chromosome 14 open reading frame 142	0.19704676	2.13813E-05
9481	SLC25A27	solute carrier family 25, member 27	0.198188373	8.25666E-05
6708	SPTA1	spectrin, alpha, erythrocytic 1 (elliptocytosis 2)	0.198230692	1.01877E-05
2936	GSR	glutathione reductase	0.199527565	1.63443E-06
10562	OLFM4	olfactomedin 4	0.199647096	1.15847E-05
7991	TUSC3	tumor suppressor candidate 3	0.200399391	3.74354E-05
10681	GNB5	guanine nucleotide binding protein (G protein), beta 5	0.201370766	5.0234E-05
23040	MYT1L	myelin transcription factor 1-like	0.204647283	8.31875E-05
22901	ARSG	arylsulfatase G	0.208725376	5.32396E-05
10279	PRSS16	protease, serine, 16 (thymus)	0.210284553	7.90389E-05
54939	COMMD4	COMM domain containing 4	0.210664422	4.94578E-05
1770	DNAH9	dynein, axonemal, heavy chain 9	0.212595043	9.92987E-06
894	CCND2	cyclin D2	0.213765457	6.53823E-05
7356	SCGB1A1	secretoglobin, family 1A, member 1 (uteroglobin)	0.214429919	7.1649E-05
283484	LOC283484	uncharacterized LOC283484	0.216069327	3.39563E-05
57650	KIAA1524	KIAA1524	0.216374441	1.19481E-05
4139	MARK1	MAP/microtubule affinity-regulating kinase 1	0.217306843	9.01463E-05
27098	CLUL1	clusterin-like 1 (retinal)	0.217430566	1.7684E-05
8301	PICALM	phosphatidylinositol binding clathrin assembly protein	0.218691398	9.96687E-06
55110	MAGOHB	mago-nashi homolog B (Drosophila)	0.21950427	5.82891E-05
163081	ZNF567	zinc finger protein 567	0.219687149	4.08174E-05
80700	UBXN6	UBX domain protein 6	0.22029261	5.64717E-05
1731	SEPT1	septin 1	0.220367831	4.45004E-05
54414	SIAE	sialic acid acetylesterase	0.222876791	6.67167E-05

1741	DLG3	discs, large homolog 3 (Drosophila)	0.222930685	8.59311E-06
152756	C4orf39	chromosome 4 open reading frame 39	0.222995615	8.84069E-06
169270	ZNF596	zinc finger protein 596	0.223311428	7.30718E-06
5567	PRKACB	protein kinase, cAMP-dependent, catalytic, beta	0.224140879	2.69271E-05
9869	SETDB1	SET domain, bifurcated 1	0.2253656	1.81497E-05
7270	TTF1	transcription termination factor, RNA polymerase I	0.226659218	2.28839E-05
2794	GNL1	guanine nucleotide binding protein-like 1	0.226797093	3.09869E-06
23332	CLASP1	Cytoplasmic linker associated protein 1	0.226910712	7.3493E-05
283310	C12orf64	chromosome 12 open reading frame 64	0.22780007	4.15357E-05
51275	C12orf47	chromosome 12 open reading frame 47	0.230619235	7.53242E-05
90317	ZNF616	zinc finger protein 616	0.230715995	9.38919E-05
284656	EPHA10	EPH receptor A10	0.230742139	1.67301E-05
160518	DENND5B	DENN/MADD domain containing 5B	0.231022895	6.26944E-05
64398	MPP5	membrane protein, palmitoylated 5 (MAGUK p55 subfamily member 5)	0.232247403	4.09094E-05
8543	LMO4	LIM domain only 4	0.232544867	4.43493E-05
1982	EIF4G2	eukaryotic translation initiation factor 4 gamma, 2	0.233795543	7.04498E-05
57528	KCTD16	potassium channel tetramerisation domain containing 16	0.234745918	5.32779E-05
63974	NEUROD6	neurogenic differentiation 6	0.237572737	6.60728E-05
51496	CTDSPL2	CTD (carboxy-terminal domain, RNA polymerase II, polypeptide A) small phosphatase like 2	0.240790778	2.02993E-05
79783	SUGCT	succinyl-CoA:glutarate-CoA transferase	0.241107758	3.62348E-05
51608	GET4	golgi to ER traffic protein 4 homolog (S. cerevisiae)	0.241383495	5.94325E-06
515	ATP5F1	ATP synthase, H+ transporting, mitochondrial Fo complex, subunit B1	0.241431174	1.40459E-05
1984	EIF5A	eukaryotic translation initiation factor 5A	0.241553721	2.19357E-06
27319	BHLHE22	basic helix-loop-helix family, member e22	0.242258502	8.42321E-05
147645	VSIG10L	V-set and immunoglobulin domain containing 10 like	0.242683646	8.85379E-05
56993	TOMM22	translocase of outer mitochondrial membrane 22 homolog (yeast)	0.243163969	5.41726E-06
56052	ALG1	asparagine-linked glycosylation 1, beta-1,4-mannosyltransferase homolog (S. cerevisiae)	0.247020898	1.48808E-05
151195	CCNYL1	cyclin Y-like 1	0.247698446	4.9648E-05
22948	CCT5	chaperonin containing TCP1, subunit 5 (epsilon)	0.247856464	6.15965E-05
80224	NUBPL	nucleotide binding protein-like	0.248161621	7.20475E-05
10658	CELF1	CUGBP, Elav-like family member 1	0.248454389	9.33091E-05
9677	PPIP5K1	diphosphoinositol pentakisphosphate kinase 1	0.249381978	1.35708E-07
3382	ICA1	islet cell autoantigen 1, 69kDa	0.249762845	2.41638E-05
54148	MRPL39	mitochondrial ribosomal protein L39	0.25067943	8.7033E-05
55862	ECHDC1	enoyl CoA hydratase domain containing 1	0.251455215	4.37292E-05
642924	LINC00535	long intergenic non-protein coding RNA 535	0.252893819	6.83023E-06
7881	KCNAB1	potassium voltage-gated channel, shaker-related subfamily, beta member 1	0.253379497	1.54E-05
645191	LINGO3	leucine rich repeat and Ig domain containing 3	0.25400303	3.56459E-05
147007	TMEM199	transmembrane protein 199	0.254036375	2.23978E-06
100132884	LOC100132884	uncharacterized LOC100132884	0.2546586	8.09859E-05
101928498	LOC101928498	uncharacterized LOC101928498	0.257003889	1.71389E-06
9470	EIF4E2	eukaryotic translation initiation factor 4E family member 2	0.259272145	9.46231E-05
6945	MLX	MAX-like protein X	0.260489569	6.51217E-05
10775	POP4	processing of precursor 4, ribonuclease P/MRP subunit (S. cerevisiae)	0.260614809	7.92389E-06
286359	LOC286359	uncharacterized LOC286359	0.261719351	1.21654E-05
51105	PHF20L1	PHD finger protein 20-like 1	0.261767529	3.50347E-05
100507557	LOC100507557	uncharacterized LOC100507557	0.262771642	8.02713E-05
51111	SUV420H1	suppressor of variegation 4-20 homolog 1 (Drosophila)	0.263004871	4.22888E-05
1807	DPYS	dihydropyrimidinase	0.263121132	4.91402E-06
7336	UBE2V2	ubiquitin-conjugating enzyme E2 variant 2	0.268049344	4.64289E-05
55027	HEATR3	HEAT repeat containing 3	0.269701101	4.01842E-05
78991	PCYOX1L	prenylcysteine oxidase 1 like	0.269715877	4.43934E-05
3419	IDH3A	isocitrate dehydrogenase 3 (NAD+) alpha	0.271499204	9.95633E-05
9063	PIAS2	Protein inhibitor of activated STAT, 2	0.271684484	4.41772E-05
1084	CEACAM3	carcinoembryonic antigen-related cell adhesion molecule 3	0.271821968	5.7677E-05
844	CASQ1	calsequestrin 1 (fast-twitch, skeletal muscle)	0.271852315	3.03481E-05
10237	SLC35B1	solute carrier family 35, member B1	0.272823999	7.92528E-05
65244	SPATS2	spermatogenesis associated, serine-rich 2	0.273482519	6.42035E-05
91147	TMEM67	transmembrane protein 67	0.273483738	8.15071E-05
285440	CYP4V2	cytochrome P450, family 4, subfamily V, polypeptide 2	0.27381825	1.39834E-06
1785	DNM2	dynamin 2	0.274895786	2.11917E-05
29761	USP25	ubiquitin specific peptidase 25	0.274909483	3.05449E-05
11218	DDX20	DEAD (Asp-Glu-Ala-Asp) box polypeptide 20	0.275382525	1.28363E-05
255352	C10orf93	chromosome 10 open reading frame 93	0.275661283	8.60497E-05
79183	TTPAL	tocopherol (alpha) transfer protein-like	0.27810543	1.92919E-05
22869	ZNF510	zinc finger protein 510	0.278916363	3.72453E-05
5599	MAPK8	mitogen-activated protein kinase 8	0.280823911	1.47051E-05
7024	TFCP2	transcription factor CP2	0.281014494	4.15538E-05
11130	ZWINT	ZW10 interactor	0.281253181	2.7161E-07
9788	MTSS1	metastasis suppressor 1	0.282059367	5.65722E-05
129285	KLRAQ1	KLRAQ motif containing 1	0.286866593	3.05391E-06
1662	DDX10	DEAD (Asp-Glu-Ala-Asp) box polypeptide 10	0.287532559	3.55595E-05
11034	DSTN	Destrin (actin depolymerizing factor)	0.287674004	2.96698E-05
6880	TAF9	TAF9 RNA polymerase II, TATA box binding protein (TBP)-associated factor, 32kDa	0.289512359	7.06464E-05
286144	C8orf83	chromosome 8 open reading frame 83	0.291989129	6.82599E-05
54039	PCBP3	poly(rC) binding protein 3	0.292139459	9.23825E-05
5645	PRSS2	protease, serine, 2 (trypsin 2)	0.29347403	3.63464E-05
84451	KIAA1804	mixed lineage kinase 4	0.293869593	2.47971E-05
339965	CCDC158	coiled-coil domain containing 158	0.294669218	9.13572E-06
22949	PTGR1	prostaglandin reductase 1	0.295070422	2.10552E-05

9802	DAZAP2	DAZ associated protein 2	0.295709943	6.90278E-05
2170	FABP3	fatty acid binding protein 3, muscle and heart (mammary-derived growth inhibitor)	0.298322552	9.02347E-05
84557	MAP1LC3A	microtubule-associated protein 1 light chain 3 alpha	0.299830163	1.36819E-06
113174	SAAL1	serum amyloid A-like 1	0.30157444	7.69427E-06
56256	SERTAD4	SERTA domain containing 4	0.302069796	5.66397E-05
84798	C19orf48	chromosome 19 open reading frame 48	0.304409689	2.50785E-05
9577	BRE	brain and reproductive organ-expressed (TNFRSF1A modulator)	0.306075086	9.92354E-05
84900	RNFT2	ring finger protein, transmembrane 2	0.306171729	4.52424E-05
261726	TIPRL	TIP41, TOR signaling pathway regulator-like (S. cerevisiae)	0.307187952	4.81912E-05
166379	BBS12	Bardet-Biedl syndrome 12	0.307793398	1.64386E-05
11144	DMC1	DMC1 dosage suppressor of mck1 homolog, meiosis-specific homologous recombination (yeast)	0.309237619	5.48091E-06
55216	C11orf57	chromosome 11 open reading frame 57	0.311048275	1.04698E-05
23329	TBC1D30	TBC1 domain family, member 30	0.31573145	5.64438E-05
8745	ADAM23	ADAM metallopeptidase domain 23	0.317318146	4.87044E-05
84142	FAM175A	family with sequence similarity 175, member A	0.318203448	2.67463E-05
55786	ZNF415	zinc finger protein 415	0.320643226	2.88337E-05
401442	LOC401442	uncharacterized LOC401442	0.323075963	3.91753E-05
80820	EEPD1	endonuclease/exonuclease/phosphatase family domain containing 1	0.323089105	5.1179E-05
23185	LARP4B	La ribonucleoprotein domain family, member 4B	0.323170088	4.43628E-05
92	ACVR2A	activin A receptor, type IIA	0.327396714	6.95311E-06
154215	NKAIN2	Na+/K+ transporting ATPase interacting 2	0.327625458	7.47158E-05
162394	SLFN5	schlafen family member 5	0.327952908	9.92377E-05
79644	SRD5A3	steroid 5 alpha-reductase 3	0.328233834	5.36134E-05
135295	SRSF12	serine/arginine-rich splicing factor 12	0.330721489	3.56501E-05
8417	STX7	syntaxin 7	0.331856143	4.05275E-05
8882	ZNF259	zinc finger protein 259	0.332476459	4.81463E-06
11143	MYST2	MYST histone acetyltransferase 2	0.332553475	7.67737E-05
441061	MARCH11	membrane-associated ring finger (C3HC4) 11	0.333104755	3.48392E-05
168667	BMPER	BMP binding endothelial regulator	0.333885173	6.68363E-05
151194	FAM119A	family with sequence similarity 119, member A	0.334401486	8.06696E-05
9329	GTF3C4	general transcription factor IIIC, polypeptide 4, 90kDa	0.3354717	3.24135E-05
60526	C2orf43	chromosome 2 open reading frame 43	0.336549456	1.18393E-05
8498	RANBP3	RAN binding protein 3	0.336838662	4.15908E-05
8798	DYRK4	dual-specificity tyrosine-(Y)-phosphorylation regulated kinase 4	0.342786093	4.27269E-05
8396	PIP4K2B	phosphatidylinositol-5-phosphate 4-kinase, type II, beta	0.346321233	7.98368E-05
26002	MOXD1	monooxygenase, DBH-like 1	0.350988747	8.95184E-05
6419	SETMAR	SET domain and mariner transposase fusion gene	0.35787671	2.36463E-05
23096	IQSEC2	IQ motif and Sec7 domain 2	0.358468105	8.59694E-05
3754	KCNF1	potassium voltage-gated channel, subfamily F, member 1	0.359533309	4.95636E-05
9515	STXBP5L	syntaxin binding protein 5-like	0.360338998	8.58457E-05
47	ACLY	ATP citrate lyase	0.361128893	9.52106E-05
55973	BCAP29	B-cell receptor-associated protein 29	0.3634238	5.20388E-05
9131	AIFM1	apoptosis-inducing factor, mitochondrion-associated, 1	0.368230385	6.62156E-05
388272	C16orf87	chromosome 16 open reading frame 87	0.371197394	1.52935E-05
11285	B4GALT7	xylosylprotein beta 1,4-galactosyltransferase, polypeptide 7 (galactosyltransferase I)	0.372874153	7.20649E-06
91543	RSAD2	radical S-adenosyl methionine domain containing 2	0.383620394	8.54968E-05
23184	MESDC2	mesoderm development candidate 2	0.39605989	3.45543E-05
23041	MON2	MON2 homolog (S. cerevisiae)	0.404470706	8.63572E-05
22929	SEPHS1	selenophosphate synthetase 1	0.404606953	3.40831E-05
81545	FBXO38	F-box protein 38	0.416551062	4.34948E-05
10360	NPM3	nucleophosmin/nucleoplasmin 3	0.417979562	2.84678E-05
100303728	SLC25A5-AS1	SLC25A5 antisense RNA 1	0.436709024	8.65633E-05
8111	GPR68	G protein-coupled receptor 68	0.442242867	7.38844E-05
54884	RETSAT	retinol saturase (all-trans-retinol 13,14-reductase)	0.460077878	1.06392E-05
720	C4A	complement component 4A (Rodgers blood group)	2.992510275	8.64066E-05
9117	SEC22C	SEC22 vesicle trafficking protein homolog C (S. cerevisiae)	3.326491174	2.84927E-05
56288	PARD3	par-3 family cell polarity regulator	3.762403671	1.03744E-05
94	ACVRL1	activin A receptor type II-like 1	5.081871823	7.65974E-05
202181	LOC202181	SUMO-interacting motifs containing 1 pseudogene	6.913531993	5.09011E-06

The set of 215 DEGs in LCM-captured cortical neurons of the superior frontal gyrus of AD satisfying p-Value < 0.0001 by two-tailed t-test and fold change greater than 2 or smaller than 0.5 were extracted by microarray data analysis of GSE5281. They are listed with Entrez Gene ID, Gene Symbol, Gene Name, Fold Change, and p-Value. NEUROD6 is underlined.

Supplementary TABLE 4. The set of 131 DEGs downregulated in the hippocampal CA1 region during progression of AD identified by microarray data analysis of GSE1297

Entrez Gene ID	Gene Symbol	Gene Name	Fold Change (Severe AD vs NC)	p-Value
57172	CAMK1G	calcium/calmodulin-dependent protein kinase IG	0.119791216	0.00015044
1290	COL5A2	collagen, type V, alpha 2	0.137353439	0.003310089
7447	VSNL1	visinin-like 1	0.176659556	0.003811085
4253	CTAGE5	CTAGE family, member 5	0.187301453	0.000234565
11161	C14orf1	chromosome 14 open reading frame 1	0.200480779	0.005009542
56603	CYP26B1	cytochrome P450, family 26, subfamily B, polypeptide 1	0.209620966	0.001291311
10675	CSPG5	chondroitin sulfate proteoglycan 5 (neuroglycan C)	0.212310725	0.003058062
10368	CACNG3	calcium channel, voltage-dependent, gamma subunit 3	0.212906942	0.000645324
2115	ETV1	ets variant 1	0.22931704	0.000240241
55711	FAR2	fatty acyl CoA reductase 2	0.230090829	0.00066547
324	APC	adenomatous polyposis coli	0.240819317	0.000639442
1716	DGUOK	deoxyguanosine kinase	0.245686681	0.001149261
10197	PSME3	proteasome (prosome, macropain) activator subunit 3 (PA28 gamma; Ki)	0.251903297	0.001593785
9378	NRXN1	neurexin 1	0.260881827	0.009477858
1795	DOCK3	dedicator of cytokinesis 3	0.261184326	0.005551229
545	ATR	ataxia telangiectasia and Rad3 related	0.262908596	0.003036901
10769	PLK2	polo-like kinase 2	0.264329482	0.001588852
23026	MYO16	myosin XVI	0.264525437	0.003540514
6623	SNCG	synuclein, gamma (breast cancer-specific protein 1)	0.266115782	0.002115945
3084	NRG1	neuregulin 1	0.266870545	0.001406226
27292	DIMT1L	DIM1 dimethyladenosine transferase 1-like (S. cerevisiae)	0.275042863	0.002562807
6715	SRD5A1	steroid-5-alpha-reductase, alpha polypeptide 1 (3-oxo-5 alpha-steroid delta 4-dehydrogenase alpha 1)	0.282086506	0.001299877
9331	B4GALT6	UDP-Gal:betaGlcNAc beta 1,4- galactosyltransferase, polypeptide 6	0.284246871	0.005450676
11197	WIF1	WNT inhibitory factor 1	0.290913711	0.001653579
286	ANK1	ankyrin 1, erythrocytic	0.295816536	0.009830239
3040	HBA2	hemoglobin, alpha 2	0.299863012	0.004878631
5132	PDC	phosducin	0.301022115	0.003171501
55521	TRIM36	tripartite motif-containing 36	0.303428631	0.002870895
11178	LZTS1	leucine zipper, putative tumor suppressor 1	0.318678801	0.000656311
3043	HBB	hemoglobin, beta	0.320523818	0.005373169
55312	RFK	riboflavin kinase	0.326351561	0.000250327
4741	NEFM	neurofilament, medium polypeptide	0.334625635	0.001051236
5274	SERPINI1	serpin peptidase inhibitor, clade I (neuroserpin), member 1	0.337526915	0.006332563
9409	PEX16	peroxisomal biogenesis factor 16	0.338900118	0.000820529
9079	LDB2	LIM domain binding 2	0.340999225	0.003326867
1268	CNR1	cannabinoid receptor 1 (brain)	0.344653589	0.008057005
5579	PRKCB	protein kinase C, beta	0.345004889	0.002186833
80742	PRR3	proline rich 3	0.357389253	0.009060604
100996492	LINC01314	long intergenic non-protein coding RNA 1314	0.366700351	0.009201076
63982	ANO3	anoctamin 3	0.372084936	0.008600399
114884	OSBPL10	oxysterol binding protein-like 10	0.377322152	1.56064E-05
6566	SLC16A1	solute carrier family 16, member 1 (monocarboxylic acid transporter 1)	0.377389214	0.008899999
6045	RNF2	ring finger protein 2	0.379240231	0.00262118
51099	ABHD5	abhydrolase domain containing 5	0.38334089	0.005300446
9699	RIMS2	regulating synaptic membrane exocytosis 2	0.38701032	0.000579041
81831	NETO2	neuropilin (NRP) and tolloid (TLL)-like 2	0.393977566	0.000965658
9143	SYNGR3	synaptogyrin 3	0.397630789	0.005052114
1875	E2F5	E2F transcription factor 5, p130-binding	0.408606441	0.009410234
8548	BLZF1	basic leucine zipper nuclear factor 1	0.413990554	0.003290634
440270	GOLGA8B	golgin A8 family, member B	0.422538873	0.004711934
23236	PLCB1	phospholipase C, beta 1 (phosphoinositide-specific)	0.43589904	0.001873881
2890	GRIA1	glutamate receptor, ionotropic, AMPA 1	0.436494345	0.009682402
93408	MYL10	myosin, light chain 10, regulatory	0.439384927	0.008276491
5937	RBMS1	RNA binding motif, single stranded interacting protein 1	0.441701035	0.009373718
8470	SORBS2	sorbin and SH3 domain containing 2	0.444625873	0.000784413
64771	C6orf106	chromosome 6 open reading frame 106	0.446598876	0.009694381
27324	TOX3	TOX high mobility group box family member 3	0.450425735	0.002724595
5994	RFXAP	regulatory factor X-associated protein	0.451935762	0.006501679
6000	RGS7	regulator of G-protein signaling 7	0.451971533	0.006045425
138046	RALYL	RALY RNA binding protein-like	0.453463038	0.001029166
53335	BCL11A	B-cell CLL/lymphoma 11A (zinc finger protein)	0.457377016	0.004390769
8604	SLC25A12	solute carrier family 25 (mitochondrial carrier, Aralar), member 12	0.457499507	0.00019237
22986	SORCS3	sortilin-related VPS10 domain containing receptor 3	0.459432107	0.003125207
5530	PPP3CA	protein phosphatase 3, catalytic subunit, alpha isozyme	0.461727097	0.001385837
23392	KIAA0368	KIAA0368	0.462082599	0.003454136
1020	CDK5	cyclin-dependent kinase 5	0.464742579	0.00340998
5577	PRKAR2B	protein kinase, cAMP-dependent, regulatory, type II, beta	0.465879457	0.002607569

9856	KIAA0319	KIAA0319	0.466821918	0.003104747
9750	FAM65B	family with sequence similarity 65, member B	0.469502963	0.003648222
57863	CADM3	cell adhesion molecule 3	0.470400343	0.001417647
4121	MAN1A1	mannosidase, alpha, class 1A, member 1	0.470575788	0.007786277
23281	MTUS2	microtubule associated tumor suppressor candidate 2	0.475295934	0.007578628
23167	EFR3A	EFR3 homolog A (S. cerevisiae)	0.480846757	0.004322177
3039	HBA1	hemoglobin, alpha 1	0.481011431	0.006622563
4907	NT5E	5'-nucleotidase, ecto (CD73)	0.481199125	0.008511804
4830	NME1	non-metastatic cells 1, protein (NM23A) expressed in	0.482930377	0.004989266
3048	HBG2	hemoglobin, gamma G	0.483511346	0.001076114
3751	KCND2	potassium voltage-gated channel, Shal-related subfamily, member 2	0.489787298	0.004105453
27324	TOX3	TOX high mobility group box family member 3	0.490769258	0.005222024
10129	FRY	furry homolog (Drosophila)	0.49309084	0.00556954
3191	HNRNPL	heterogeneous nuclear ribonucleoprotein L	0.499634764	0.003832929
8502	PKP4	plakophilin 4	0.50203014	0.001264843
3708	ITPR1	inositol 1,4,5-triphosphate receptor, type 1	0.50283747	0.002087049
6722	SRF	serum response factor (c-fos serum response element-binding transcription factor)	0.507259691	0.009083026
9071	CLDN10	claudin 10	0.512449482	0.005138908
2335	FN1	fibronectin 1	0.51287327	0.003377587
9922	IQSEC1	IQ motif and Sec7 domain 1	0.517453432	0.000135105
79567	FAM65A	family with sequence similarity 65, member A	0.517677872	0.005148038
23157	SEPT6	septin 6	0.521243095	1.94717E-05
6310	ATXN1	ataxin 1	0.529973111	0.000693097
1107	CHD3	chromodomain helicase DNA binding protein 3	0.531318284	0.002487892
79101	TAF1D	TATA box binding protein (TBP)-associated factor, RNA polymerase I, D, 41kDa	0.534934964	0.006396605
9821	RB1CC1	RB1-inducible coiled-coil 1	0.535541656	0.003745766
55757	UGGT2	UDP-glucose glycoprotein glucosyltransferase 2	0.535558689	0.001405379
29114	TAGLN3	transgelin 3	0.536481218	0.00215522
9590	AKAP12	A kinase (PRKA) anchor protein 12	0.538894732	8.64065E-05
57496	MKL2	MKL/myocardin-like 2	0.541306767	0.009963246
53353	LRP1B	low density lipoprotein receptor-related protein 1B	0.541847962	0.004672477
23231	SEL1L3	sel-1 suppressor of lin-12-like 3 (C. elegans)	0.542192244	0.007063601
56943	ENY2	enhancer of yellow 2 homolog (Drosophila)	0.544071595	0.009527721
79762	C1orf115	chromosome 1 open reading frame 115	0.546532572	0.005130192
10846	PDE10A	phosphodiesterase 10A	0.549157947	0.002263882
1349	COX7B	cytochrome c oxidase subunit VIIb	0.553088625	0.004051697
65981	CAPRIN2	caprin family member 2	0.555441174	0.00066017
7534	YWHAZ	tyrosine 3-monooxygenase/tryptophan 5-monooxygenase activation protein, zeta polypeptide	0.556167619	0.006101886
1337	COX6A1	cytochrome c oxidase subunit VIa polypeptide 1	0.557465998	0.001751281
9353	SLIT2	slit homolog 2 (Drosophila)	0.558570719	0.009460307
26507	CNNM1	cyclin M1	0.5599908	0.005691077
93664	CADPS2	Ca++-dependent secretion activator 2	0.560315994	0.002336932
509	ATP5C1	ATP synthase, H+ transporting, mitochondrial F1 complex, gamma polypeptide 1	0.563516199	0.005837604
491	ATP2B2	ATPase, Ca++ transporting, plasma membrane 2	0.564364625	0.008806963
91851	CHRDL1	chordin-like 1	0.565566821	0.003016121
56252	YLPM1	YLP motif containing 1	0.566024088	0.00755332
377	ARF3	ADP-ribosylation factor 3	0.567332148	0.005452724
4862	NPAS2	neuronal PAS domain protein 2	0.567397135	0.007890075
63974	NEUROD6	neurogenic differentiation 6	0.568549476	0.007199422
9452	ITM2A	integral membrane protein 2A	0.56961321	0.001581406
5162	PDHB	pyruvate dehydrogenase (lipoamide) beta	0.573206217	0.006116724
4714	NDUFB8	NADH dehydrogenase (ubiquinone) 1 beta subcomplex, 8, 19kDa	0.578140448	0.001083971
22941	SHANK2	SH3 and multiple ankyrin repeat domains 2	0.580635291	0.006476589
6529	SLC6A1	solute carrier family 6 (neurotransmitter transporter, GABA), member 1	0.582511461	0.004142352
58189	WFDC1	WAP four-disulfide core domain 1	0.582843283	0.002318206
1008	CDH10	cadherin 10, type 2 (T2-cadherin)	0.584467028	0.002335459
23518	R3HDM1	R3H domain containing 1	0.590781827	0.009081363
9994	CASP8AP2	caspase 8 associated protein 2	0.591049072	0.002678888
9168	TMSB10	thymosin beta 10	0.591288491	0.000133143
2744	GLS	glutaminase	0.591660135	0.007897416
2915	GRM5	glutamate receptor, metabotropic 5	0.592186084	0.00764757
576	BAI2	brain-specific angiogenesis inhibitor 2	0.593582138	0.008425238
64788	LMF1	lipase maturation factor 1	0.596678442	0.004542002
9847	KIAA0528	KIAA0528	0.599243843	0.006295878

The set of 131 DEGs downregulated in the hippocampal CA1 region among incipicent, moderate, and severe AD and NC groups by one-way ANOVA satisfying p-Value < 0.01 and fold change of severe AD versus NC smaller than 0.6 were extracted by microarray data analysis of GSE1297. They are listed with Entrez Gene ID, Gene Symbol, Gene Name, Fold Change, and p-Value. NEUROD6 is underlined.

Supplementary TABLE 5. The set of 31 DEGs downregulated in the hippocampus of Japanese AD patients identified by microarray data analysis of GSE36980

Entrez Gene ID	Gene Symbol	Gene Name	Fold Change (AD vs non-AD)	p-Value
4233	MET	met proto-oncogene (hepatocyte growth factor receptor)	0.347976759	0.001202064
63974	NEUROD6	neurogenic differentiation 6	0.433171474	0.001616741
5816	PVALB	parvalbumin	0.457320297	0.001537504
4885	NPTX2	neuronal pentraxin II	0.486879325	0.000695301
10368	CACNG3	calcium channel, voltage-dependent, gamma subunit 3	0.496685808	0.004499281
3208	HPCA	hippocalcin	0.504149269	0.000333079
5176	SERPINF1	serpin peptidase inhibitor, clade F (alpha-2 antiplasmin, pigment epithelium derived factor), member 1	0.522226034	0.00019849
348980	HCN1	hyperpolarization activated cyclic nucleotide-gated potassium channel 1	0.523810594	0.004508668
376267	RAB15	RAB15, member RAS onocogene family	0.537387889	0.001299325
3595	IL12RB2	interleukin 12 receptor, beta 2	0.538167968	0.00328347
2675	GFRA2	GDNF family receptor alpha 2	0.539054643	0.001333403
407052	MIR95	microRNA 95	0.542735216	0.001639895
161145	TMEM229B	chromosome 14 open reading frame 83	0.545710178	0.000720208
5774	PTPN3	protein tyrosine phosphatase, non-receptor type 3	0.54910964	0.001537752
23349	KIAA1045	KIAA1045	0.55244187	0.003426591
8507	ENC1	ectodermal-neural cortex (with BTB-like domain)	0.559652363	0.003288053
266722	HS6ST3	heparan sulfate 6-O-sulfotransferase 3	0.560440342	0.001753288
53335	BCL11A	B-cell CLL/lymphoma 11A (zinc finger protein)	0.56767029	0.002900497
6304	SATB1	SATB homeobox 1	0.570165581	0.001900759
64409	WBSCR17	Williams-Beuren syndrome chromosome region 17	0.572117803	0.003646413
84894	LINGO1	leucine rich repeat and Ig domain containing 1	0.574936403	0.000424171
84525	HOPX	HOP homeobox	0.577157339	0.000987151
2903	GRIN2A	glutamate receptor, ionotropic, N-methyl D-aspartate 2A	0.581203611	0.002390123
5138	PDE2A	phosphodiesterase 2A, cGMP-stimulated	0.583469267	0.002990823
51299	NRN1	neuritin 1	0.590013553	0.001828335
125113	KRT222	keratin 222 pseudogene	0.592296133	0.004941811
1428	CRYM	crystallin, mu	0.594516424	0.002824762
646113	LINC00643	long intergenic non-protein coding RNA 643	0.59477003	0.004445109
5923	RASGRF1	Ras protein-specific guanine nucleotide-releasing factor 1	0.59493107	0.004924286
221692	PHACTR1	phosphatase and actin regulator 1	0.597310446	0.002464967
23467	NPTXR	neuronal pentraxin receptor	0.597403904	4.42886E-05

The set of 31 DEGs downregulated in the hippocampus of Japanese AD patients satisfying p-Value < 0.005 by two-tailed t-test and fold change smaller than 0.6 were extracted by microarray data analysis of GSE36980. They are listed with Entrez Gene ID, Gene Symbol, Gene Name, Fold Change, and p-Value. NEUROD6 is underlined.
